# Polyelectrolyte-like Behavior, pH-Dependent Self-Assembly, and Emulsion Stabilizing Properties of a Model Surfactant-like Peptide Bearing Pyridine Groups

**DOI:** 10.1021/acs.biomac.6c00476

**Published:** 2026-06-11

**Authors:** Valeria Castelletto, Jani Seitsonen, Ian W. Hamley

**Affiliations:** † School of Chemistry, Food Biosciences and Pharmacy, 6816 University of Reading, Whiteknights, Reading RG6 6AD, U.K.; ‡ Nanomicroscopy Center, 174277 Aalto University, Puumiehenkuja 2, FIN-02150 Espoo, Finland

## Abstract

A surfactant-like peptide (SLP) bearing six non-native 3-(4-pyridyl)-l-alanine (Pal) residues and a C-terminal arginine residue, Pal_6_R, is shown to exhibit pH-dependent self-assembly which arises from the acid–base properties of the Pal residue (p*K*_a_ ∼ 5). At a native pH of 2.4, “polyelectrolyte” correlation hole scattering is observed due to the electrostatic repulsion of highly charged molecules. The scaling of the domain size with concentration agrees with theoretical predictions for weakly charged flexible polyelectrolytes in a semidilute solution. In contrast, twisted nanotapes are observed at pH 7. The nanotapes are shown to comprise β-sheet structures packed in interdigitated bilayers. Atomistic molecular dynamics (MD) simulations confirmed the bilayer structure of the nanotapes, with extensive hydrogen bonding, and a twisting tendency. The novel SLP can stabilize water-in-oil emulsions at pH 7, forming β-sheet bilayer structures at the water droplet interface. Pal_6_R represents a model polyelectrolyte system with additional self-assembly and emulsion stabilization properties at neutral pH.

## Graphical abstract

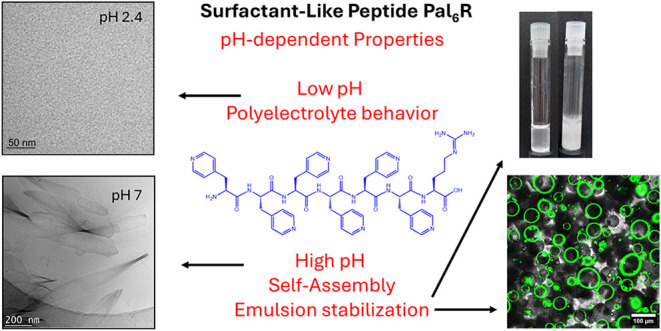

## Introduction

Surfactant-like peptides (SLPs) are a type of peptide amphiphile (PA) comprising a block of hydrophobic residues that forms the surfactant tail, which is attached to one or more charged residues serving as the headgroup.^1–3^ They have a diversity of roles as antimicrobials,^4–8^ emulsion stabilizers,^9^ membrane protein stabilizers,^10^ and so on.^3,11^

SLPs are known to self-assemble into various nanostructures depending on their sequence and the solution conditions (pH, presence of salt, etc.). Extended β-sheet structures such as fibrils or nanotapes/nanosheets are most commonly observed, as reported, for example, for SLPs such as A_6_R^12^ (here single letter codes are used as an abbreviation for peptides such as Ala_6_-Arg), A_9_R,^9^ A_12_R_2_,^13^ RA_9_R^5^ or R_3_F_3_ and R_4_F_4_,^8^ while others, including A_6_K,^14,15^ A_6_R,^16^ and R_3_L_12_ ^17,18^ form nanotubes under appropriate conditions in an aqueous solution. Micelle formation by nonlipidated peptides is observed less often, although the tetrapeptide WRWR forms micelle-like structures at low pH.^19^

The stabilization of emulsions and microemulsions is of interest in the formulation of personal care, food, and other consumer goods products, and there is increasing interest in novel stabilizers that use biocompatible compounds. Recently, peptides have been shown to be capable of stabilizing emulsions. In one example, the spontaneous formation of oil-in-water emulsions was observed in the presence of Fmoc-dipeptides (Fmoc: fluorenylmethyloxycarbonyl) containing one N-terminal aromatic residue.^20^ The emulsion droplets were stabilized by fibril networks. The tripeptide KYF functions similarly, and it was shown that enzyme-driven emulsion stabilization was possible using alkaline phosphatase to prepare KYF in situ from a mixture containing precursor peptides with phosphorylated tyrosine.^21^ This work also showed that enzyme-driven dephosphorylation enables control of assembly kinetics and associated emulsion stabilization. We previously showed that the SLP A_9_R can stabilize water-in-oil emulsions, forming β-sheet structures at the surface of the oil droplets, which stabilize the emulsion.^9^ At pH 8, it was also possible to de-emulsify the system using the enzyme elastase to degrade the oligo-alanine repeat sequence in the peptide.

In addition to emulsification, despite their short oligomeric nature, peptides can show, under defined conditions, some properties and self-assembly behavior reminiscent of those of polymers, such as glass formation^22–24^ or self-coacervation (liquid–liquid phase separation).^19,25,26^ SLP-like peptides with longer sequences start to behave as amphiphilic block polymers, self-assembling into different nanostructures in an aqueous solution.^27,28^ However, the potential for short peptides to behave as “polyelectrolytes” has rarely been considered. Polyelectrolytes are polymers comprising charged repeat units that have applications in personal care products or as flocculants, among others. It can be envisaged that under appropriate conditions, peptides bearing ionizable units, when highly charged, may exhibit certain polyelectrolyte characteristics. As reported herein, we unexpectedly found that a designed SLP bearing only 7 residues shows polyelectrolyte-like behavior (correlation hole scattering) under conditions where the peptide is expected to be fully charged.

There is substantial interest in the self-assembly of SLPs containing non-natural residues, as well as in the creation of nanostructures bearing functional groups such as pyridine, which is well-known, for example, to be capable of metal complexation underpinning metal–organic framework (MOF) formation.^29^ In addition, pyridine–metal coordination has been used with, for example, silver, to create antimicrobial molecules^30–32^ that exploit the known antibacterial properties of silver. A further potential application of pyridine-based molecules in complexes with metals such as copper is in catalysis, for example, of the water–gas shift reaction, which produces hydrogen while removing carbon monoxide.^33^ Here, we report on the self-assembly and conformation of NH_2_–Pal_6_R–OH (subsequently termed Pal_6_R), which contains 6 repeats of polar 3-(4-pyridyl)-l-alanine (Pal) and arginine (Arg, R) as the cationic headgroup. In addition, we exploit the molecule in demonstrating stabilization of water-in-oil emulsions under appropriate pH conditions, considering the p*K*_a_ of the pyridine groups. There are few prior studies on the self-assembly of Pal-containing peptides. In one example, the peptide Pal-LRLRLRL-Pal was designed to incorporate an alternating LR hydrophobic-charged sequence to promote β-sheet fibril formation.^34^

The incorporation of the Pal residue is expected to confer pH-dependent aggregation/self-assembly since the pyridine ring has an expected p*K*_a_ = 5. We therefore examined the peptide conformation and nanostructure at pH values below (pH 2.4, native) and above (pH 7 and pH 12) this value. We unexpectedly observed polyelectrolyte-like features at native pH 2.4, whereas at pH 7 and above, well-defined β-sheet nanotapes are revealed, as detailed below using a combination of spectroscopic, microscopic, and X-ray scattering methods. In addition, atomistic molecular dynamics (MD) simulations were performed to provide further information on the formation and stability of the nanotapes at pH 7. The pH-influenced peptide charge and conformation were further exploited toward a demonstrated application of the Pal_6_R peptide in the stabilization of water-in-oil emulsions at pH 7, whereas this was not observed at pH 2.4. Confocal microscopy and SAXS indicate that the emulsion is stabilized by β-sheet structures deposited at the water droplet surfaces.

## Methods

### Materials and Sample Preparation

The peptide was purchased from Peptide Synthetics (PPR Ltd., Fareham, UK) and supplied as the TFA salt. The molar mass measured by ESI-MS was 1063.2 g mol^–1^ (1062.2 g mol^–1^ expected). The chemical structure of Pal_6_R is shown in Scheme 1. The purity by HPLC (0.1% TFA in an acetonitrile/water gradient) was 96.7% for one batch and 96.6% for another.

**Scheme 1:**
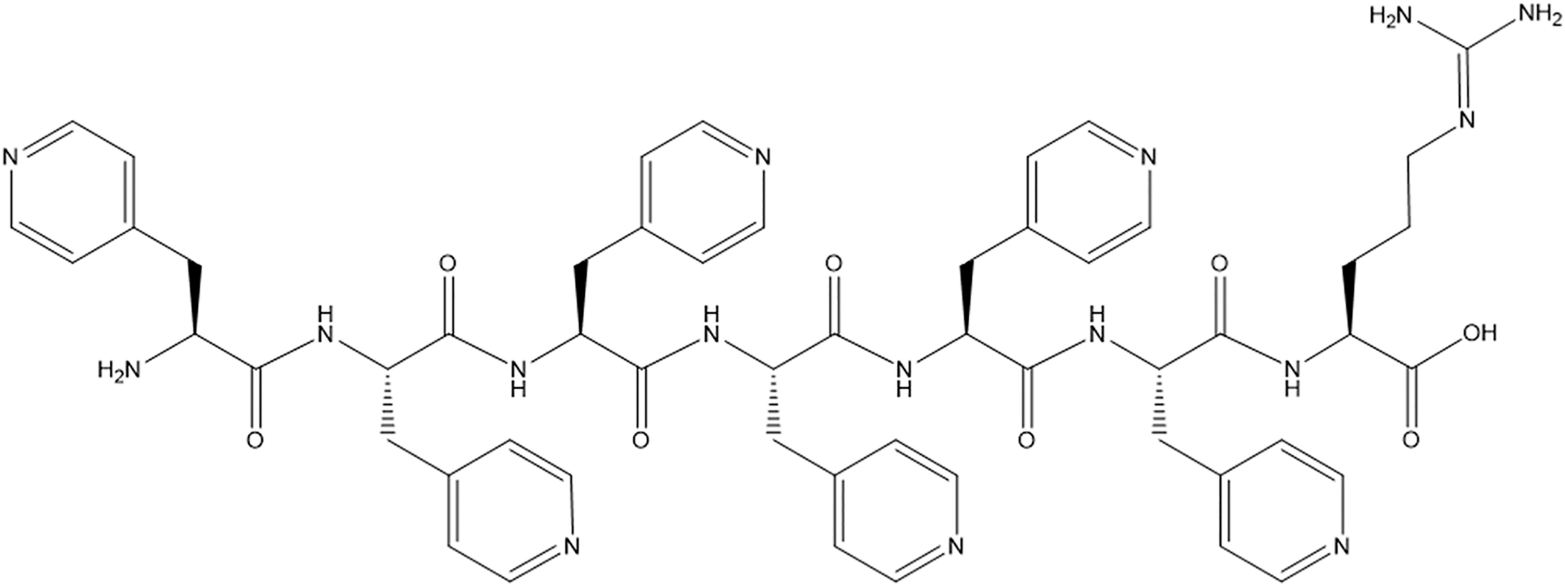
Molecular Structure of Pal_6_R

### Emulsion Preparation

Water-in-oil emulsions were prepared by choosing 1-bromohexadecane (density 1 g mL^–1^) as the oil to match the density of both phases and maximize emulsion stability.^9,35^ Emulsions were prepared by mixing 40 μL of 1 wt % Pal_6_R at native pH 2.4 or 1 wt % Pal_6_R at pH 7 with 360 μL of 1-bromohexadecane. Each mixture was vigorously stirred and vortexed at 50 °C. In this way, mixtures started from 1 wt % Pal_6_R native pH 2.4 or 1 wt % Pal_6_R pH 7 resulted in 0.1 wt % Pal_6_R pH 4 or 0.1 wt % Pal_6_R pH 7 emulsions, respectively.

A separate set of emulsions was prepared for laser scanning confocal microscopy (LSCM) experiments. These emulsions were prepared as described above, but 1 wt % Pal_6_R pH 2.4 or 1 wt % Pal_6_R pH 7 was dissolved in a 5 × 10^–3^ wt % ThT aqueous solution instead of pure water.

### Cryogenic-TEM (Cryo-TEM)

Imaging was carried out using a field-emission cryo-electron microscope (JEOL JEM-3200FSC), operating at 200 kV. Images were taken in the bright field mode and using zero loss energy filtering (omega type) with a slit width of 20 eV. Micrographs were recorded using a Gatan Ultrascan 4000 CCD camera. The specimen temperature was maintained at −187 °C during the imaging. Vitrified specimens were prepared using an automated FEI Vitrobot device using Quantifoil 3.5/1 holey carbon copper grids with a hole size of 3.5 μm. Just prior to use, grids were plasma cleaned using a Gatan Solarus 9500 plasma cleaner and then transferred into the environmental chamber of a FEI Vitrobot at room temperature and 100% humidity. Thereafter, 3 μL of sample solution was applied on the grid, and it was blotted twice for 5 s and then vitrified in a 1/1 mixture of liquid ethane and propane at a temperature of −180 °C. The grids with the vitrified sample solution were maintained at liquid nitrogen temperature and then cryo-transferred to the microscope.

### Small-Angle X-ray Scattering (SAXS)

SAXS experiments were performed on beamline B21 ^36^ at Diamond (Didcot, UK). The sample solutions were loaded into the 96-well plate of an EMBL BioSAXS robot and then injected via an automated sample exchanger into a quartz capillary (1.8 mm internal diameter) in the X-ray beam. The quartz capillary was enclosed in a vacuum chamber to avoid parasitic scattering. After the sample was injected into the capillary and reached the X-ray beam, the flow was stopped during the SAXS data acquisition. Beamline B21 operates with a fixed camera length (3.9 m) and a fixed energy (12.4 keV). The images were captured using a PILATUS 2 M detector. Data processing was performed using the dedicated beamline software ScÅtter.

### Circular Dichroism (CD) Spectroscopy

Far-UV CD spectra were collected by using a Chirascan spectropolarimeter (Applied Photophysics, Leatherhead, UK). Spectra were recorded from 180 to 280 nm. Samples were mounted in a quartz cell with detachable windows, with a 0.01 mm path length. The CD spectra from the samples were corrected by water background subtraction. CD spectra were smoothed using Chirascan software for data analysis. The residue of the calculation was chosen to oscillate around the average to avoid artifacts in the smoothed curve. CD data, measured in mdeg, were normalized to molar ellipticity using the molar concentration of the sample and the cell path length.

### Fluorescence Spectroscopy

Experiments were carried out using a 10.0 mm × 5.00 mm quartz cell in a Varian Model Cary Eclipse spectrofluorometer (Agilent, UK). The titration of fluorescent dyes thioflavin T (ThT) or 8-anilino-1-naphthalenesulfonic acid (ANS) was studied for Pal_6_R solutions at native pH 2.4 or neutral pH 7. For native solutions at pH 2.4, fluorescent dye experiments were used to detect any aggregation of Pal_6_R. For Pal_6_R solutions at pH 7, ThT and ANS were used to determine the critical aggregation concentration (CAC). Solutions were prepared with 2 × 10^–3^ wt % ANS or 5 × 10^–3^ wt % ThT. Initial concentrated aqueous solutions of peptide were titrated with 2 × 10^–3^ wt % ANS or 5 × 10^–3^ wt % ThT, and the fluorescence emission was measured for each titration point. Solutions containing ANS or ThT were excited with λ_ex_ = 356 or 440 nm, respectively.

### Fiber XRD

Measurements were performed on a peptide stalk prepared by drying a peptide solution suspended between the ends of two wax-coated capillaries. The stalk was mounted onto a four-axis goniometer of an Oxford Diffraction Gemini Ultra instrument. The sample-detector distance was 60 mm. The X-ray wavelength λ = 1.54 Å was used to calculate the scattering vector *q* = 4π sin θ/λ (2θ: scattering angle). The detector was a Sapphire CCD.

### Optical Microscopy

Experiments were performed by using an Olympus BX41 microscope. Emulsions were mounted in quartz cells with detachable windows, with a 0.2 mm path length. Solutions were placed between a microscope slide and a coverslip and observed through cross-polarizers. Images were recorded by using a GT Vision GXCAM camera.

### Laser Scanning Confocal Microscopy (LSCM)

Experiments were performed with a Nikon A1 HD25/A1R HD25 confocal microscope. Emulsions containing 5 × 10^–3^ wt % ThT were prepared as detailed above and loaded in a chamber slide with 8 wells on a glass slide. Experiments were performed using a Plan Apo 20× lens and a pinhole size of 20.43 μm. In solutions stained only with 5 × 10^–3^ wt % ThT, the ThT was excited at λ_ex_ = 488 nm. A Nikon A1 HD25/A1R HD25 microscope was also used to record images in bright-field transmission mode.

### Molecular Dynamics Simulations

Molecular dynamics simulations were performed using Gromacs^37^ (version 2024.4). The force field parameters for Pal_6_R were obtained within the charmm36_ljpme-jul2022.ff set which includes parameters for 3-(4-pyridyl)-alanine as well as arginine. To model β-sheet nanotape structures, the method described previously^38^ was adopted, in which hydrogen-bonded parallel β-sheet arrays containing 32 molecules were built, then four of these were arranged to form thin tapes containing 128 molecules. A natural packing of the Pal_6_R molecules with steric zipper stacking of the aromatic residues was noted with a pairwise opposed packing of the parallel β-sheets (SI Figure S11). Simulations were performed using the CHARMM36 force field.^39,40^ The packed molecules were placed into a rectangular box of dimensions 12 × 20 × 20 nm^3^, and the systems were solvated using TIP3P water. To model the structure corresponding to conditions at pH 7, the net charge per molecule was zero, representing the zwitterionic molecule comprising a neutral N-terminus, six uncharged 3-pyridyl-l-alanine residues, and one cationic arginine residue and a charged C-terminus. After energy minimization and 100 ps relaxation stages in the NVT ensemble, the final simulations were carried out in the NPT ensemble using a leapfrog integrator with steps of 1 fs up to 10 ns. The temperature was maintained at 298.15 K using the velocity-rescale (modified Berendsen) thermostat^41^ with a time constant of 5 ps. The pressure was maintained at 1 bar using the Parinello–Rahman barostat,^42^ and periodic boundary conditions were applied in all three dimensions. The Particle Mesh Ewald scheme^43,44^ was used for long-range electrostatics. Bonds were constrained using the LINCS algorithm,^45^ and the Verlet cutoff scheme^46^ was used. Coulomb and van der Waals cutoffs were 1.0 nm.

## Results and Discussion

We examined the self-assembly and ordering of the surfactant-like peptide Pal_6_R at two pH values. The first pH 2.4 (native) corresponds to a state in which the molecule is expected to have a charge of +8, this pH value being below the typical p*K*_a_ of the N-terminus and the arginine residues and the 3-(4-pyridyl)-l-alanine residues with an expected p*K*_a_ ∼ 5 of the pyridine rings^34,47^ and below the expected p*K*_a_ of the C-terminus. In contrast, at pH 7, the expected charge is zero, and the peptide will be zwitterionic with a charged Arg residue and C-terminus. This is expected to lead to significant differences in aggregation/self-assembly behavior. In addition, this property was exploited in the development of a pH-responsive peptide-based emulsion stabilizer. The measured titration curve for a 1.2 wt % solution of Pal_6_R upon addition of NaOH is shown in SI Figure S1 along with a calculated titration curve using the Henderson–Hasselbalch equation with the indicated assumed p*K*_a_ values for the titratable groups (all residues plus both termini) The agreement is reasonable in terms of the rough shape of the curve, but the experimental curve does not show all of the discrete p*K*_a_ inflection points predicted from the model and there are differences especially at low pH. This is probably due to the simplified nature of the Henderson–Hasselbalch approach (applicable to dilute solutions of noninteracting ionizable molecules), the estimated nature of the p*K*_a_ values, and the assumption that the p*K*_a_ values of the pyridyl groups are all of the same, which will not be the case in practice due to the effects of adjacent charged groups.

Small-angle X-ray scattering (SAXS) experiments revealed that under conditions where the peptide is highly charged at native pH 2.4, a characteristic peak is present in the data, the position and intensity of which are concentration-dependent. The data are shown in Figure 1a, which also indicates the shift in the peak maximum position q* to higher wavenumbers as the concentration increases. The q* value was quantified via fitting to the high *q* part of the data using a Lorentzian function, the fit parameters being listed in SI Table S1. The concentration-dependent increase in q* corresponds to a reduction in the associated correlation length *d* = 2π/q*, which is plotted versus concentration on a double-logarithmic scale in Figure 1b. This reveals a power-law-like scaling *d* ∼ *c* ^–(0.277 ± 0.010)^. This behavior is reminiscent of that observed for polyelectrolyte solutions, which exhibit a concentration-dependent “correlation hole” peak. The corresponding correlation length relates to the screened repulsion zone around a charged chain. In a semidilute salt-free solution of a weakly charged polyelectrolyte, the expected scaling of q* within the random phase approximation (RPA) is *d* ∼ *c* ^–1/4^.^48–52^ Our data is close to this prediction, although the obtained exponent (−0.277 ± 0.010) lies slightly outside the uncertainty from −0.25 (however, it is within three standard deviations). The observed behavior can be clearly contrasted with the scaling laws predicted for rod-like polyelectrolytes in semidilute solution *d* ∼ *c* ^–1/2^,^50,53–55^ or dilute polyelectrolyte solutions *d* ∼ *c* ^–1/3^, as shown in Figure 1b.^50,55–57^ Confirmation of the polyelectrolyte-like nature of the scattering peak is provided by SAXS data measured for concentrated NaCl solutions for which the “correlation hole” peak is absent (Figure 1c). This is due to the salt screening of the electrostatic repulsion between the charged peptide chains.

**Figure 1:**
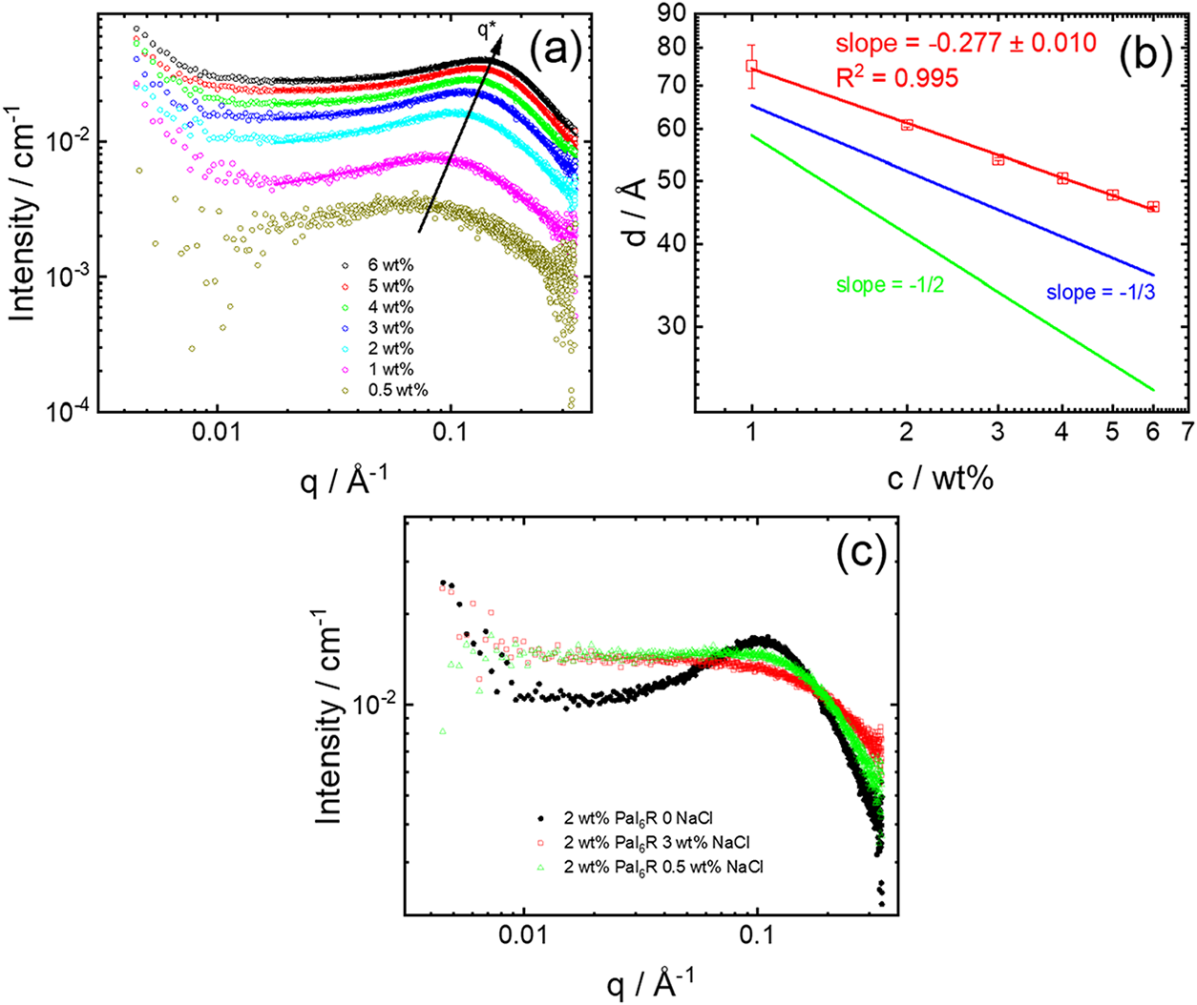
(a) Open symbols – SAXS data for Pal_6_R at native pH 2.4 at the concentrations indicated. For ease of visualization, only every 3rd data point is plotted. The lines at high *q* are peak fits used to determine q* using a Lorentzian function with sloping background. (b) Log–log plot showing power-law dependence of characteristic domain spacing *d* = 2π/q* on concentration with fitted slope, in comparison to other scaling laws discussed in the text. (c) Data for 2 wt % Pal6R in the absence and presence of salt at the concentrations indicated (3 wt % NaCl is equivalent to 0.6 M). For ease of visualization, only every 3rd data point is shown.

We investigated the conformation of Pal_6_R at native pH 2.4 via circular dichroism (CD) spectroscopy. The spectrum at pH 2.4 presented in Figure 2 is characteristic of a disordered structure, with an enhanced peak at 219 nm, assigned to the absorbance of the aromatic pyridine group. The CD indicates that the peptide has an unordered conformation lacking a defined secondary structure in the “polyelectrolyte” state. We also examined whether there is any critical concentration for the formation of polyelectrolyte clusters using fluorescence probe experiments with 8-anilino-1-naphthalenesulfonic acid (ANS), which is sensitive to the formation of hydrophobic domains.^58,59^ The concentration dependence of the ANS fluorescence peak intensity normalized to that in the solution without peptide (*I*/*I*_0_) is shown in SI Figure S2 (original spectra in SI Figure S3). It shows a continuous decrease with increased peptide concentration, which is not consistent with the behavior of a system showing any critical aggregation concentration over the range probed. At a higher concentration, above 0.15 wt %, the fluorescence signal saturates due to the saturation of binding for relative concentrations [Pal_6_R]/[ANS] > 21 (ratio of concentrations in wt %). An additional experiment using thioflavin T (ThT), a probe for amyloid β-sheet structure,^58,60^ did not provide any emission fluorescence above that of the probe in water, also indicating the lack of defined aggregation at pH 2.4.

**Figure 2:**
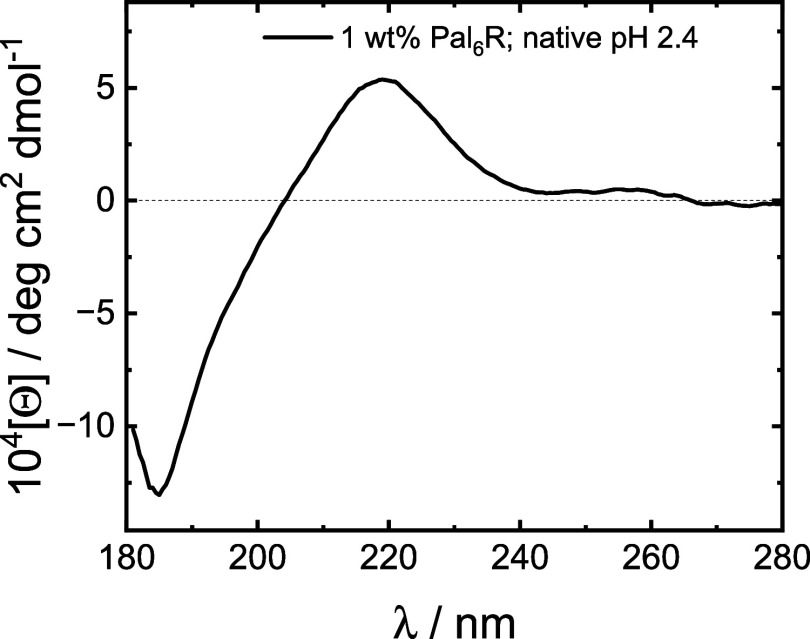
Circular dichroism spectrum for 1 wt % solution at pH 2.4.

Having established that Pal_6_R shows polyelectrolyte-like characteristics at pH 2.4, we next examined the self-assembly of Pal_6_R at higher pH, considering that under these conditions, the peptide will have a zwitterionic nature, considering the charged C-terminus and adjacent R residue. The cryo-TEM image for a 1 wt % sample at pH 7 in Figure 3a shows that Pal_6_R forms twisted nanotape structures under these conditions. Additional cryo-TEM images showing these structures are provided in SI Figure S4. A layer structure is confirmed by the SAXS data in Figure 3b, which shows (for a 2 wt % solution) a small broad Bragg peak centered at *q* = 0.195 Å^–1^ (SI Table S2) corresponding to a lamellar spacing *d* = 32 Å. The SAXS data at pH 12 shows a sharper Bragg peak at *q* = 0.201 Å^–1^ (*d* = 31.3 Å). Considering an antiparallel β-sheet, the expected strand length^61^ for a heptameric peptide would be 7 × 3.4 Å = 32 Å. The measured *d*-spacing suggests that Pal_6_R forms an interdigitated bilayer structure with Arg residues at each surface (this was used as a starting point for MD simulations discussed below, see also SI Figure S11). This was further modeled by atomistic MD simulations as described below. The SAXS data in Figure 3b was modeled, accounting for the power law intensity slope at low *q* and the peak at high *q* represented by a Lorentzian function. The fit parameters are listed in SI Table S2. Confirmation of a lamellar structure was also provided by polarized optical microscopy imaging of a Pal_6_R solution (SI Figure S5), which shows spherulite-like structures that are characteristic of radially grown lamellar structures.^62–64^

**Figure 3:**
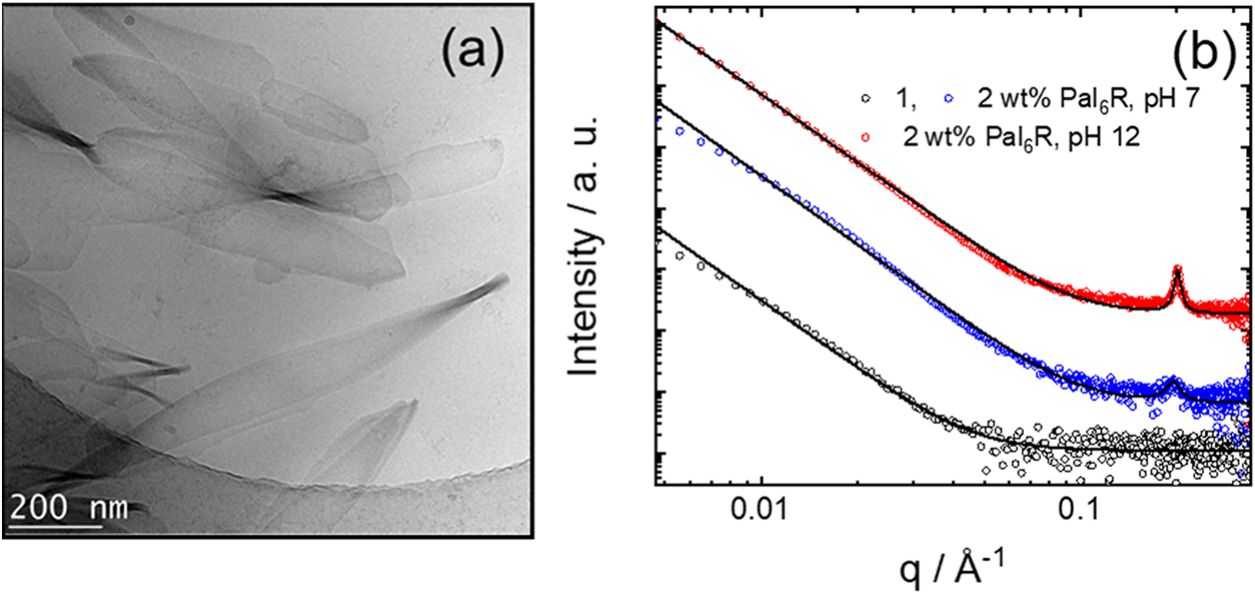
Self-assembly of Pal_6_R at pH 7. (a) Cryo-TEM image from 1 wt % solution, (b) SAXS data under the conditions indicated; open symbols: measured data; solid lines: fitted intensity profiles as described in the text (fit parameters in SI Table S2). For ease of visualization, data has been vertically shifted.

To investigate aggregation at pH 7, fluorescence probe assays were performed with both ANS and ThT. The data in Figure 4a show the fluorescence intensity versus concentration from assays using both dyes, and discontinuities in the gradient are observed for both at a similar concentration, which locates a critical aggregation concentration, CAC = (0.065 ± 0.005) wt %. The original fluorescence spectra are presented in SI Figures S6 and S7. The coincidence of the CAC from the ThT and ANS fluorescence assays suggests that β-sheet formation occurs concomitantly with the formation of hydrophobic domains (presumed to be the hydrophobic Pal_6_R domains within bilayers, discussed in more detail below). The conformation of Pal_6_R at pH 7 was probed using CD spectroscopy. The spectrum shown in Figure 4b shows a strong negative minimum at 228 nm, assigned as a red-shifted β-sheet feature. The red shift may be due to aggregation into extended nanostructures (with light scattering)^65^ and/or the peak may be shifted due to the aforementioned closely located pyridine aromatic ring peak at 219 nm. CD was complemented by XRD on a stalk prepared from a 2 wt % solution. The XRD intensity profile shown in Figure 4c exhibits a series of reflections, including strong peaks at 4.6 and 4 Å, which are assigned to β-strand spacings along with longer spacings from the stacking of the β-strands in the β-sheets and a 3.2 Å spacing from the repeat distance along the β-strand. The other peaks can tentatively be assigned to the bilayer spacing (17 and 12 Å, close to *d*/2 and *d*/3) and other periodicities in the β-strand (6.5 and 7.2 Å),^66,67^ although there are not enough peaks to enable a unit cell determination and full peak assignment.

**Figure 4:**
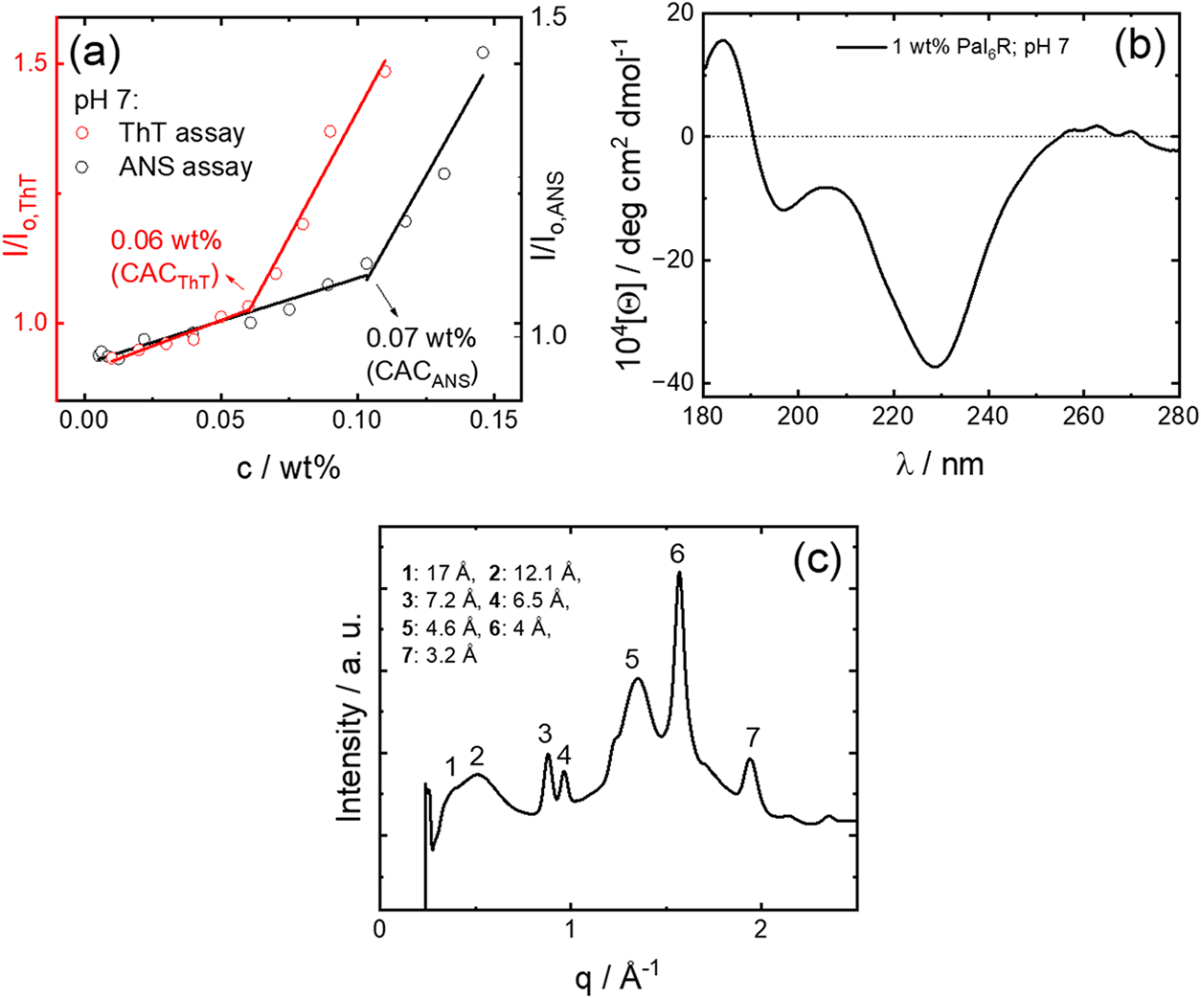
Aggregation and conformation of Pal_6_R at pH 7. (a) Determination of critical aggregation concentration (CAC) from the intensity of the ANS (ThT) peak fluorescence *I*_ANS_ (*I*_ThT_) relative to that of the solution without peptide *I*_0,ANS_ (*I*_0,ThT_). (b) Circular dichroism spectrum at conditions indicated. (c) XRD spectra stalk dried from a 2 wt % solution of Pal_6_R at pH 7.

The reversibility of the transition between the β-sheet nanotape structure at pH 7 and the polyelectrolyte-like unordered structure at native pH 2.4 was examined using CD spectroscopy. Spectra were measured upon reduction from the initial pH 7 and are shown in SI Figure S8. The spectra show the recovery of the disordered spectrum at pH 2.4 with a high similarity to the spectrum measured directly at pH 2.4, shown in Figure 2, and this is also the case at pH 4.5. The spectra show that the transition from the disordered structure to the β-sheet structure occurs close to pH 5, near the expected p*K*_a_ of the pyridyl groups. This was further confirmed by cryo-TEM images (SI Figure S9), which show a twisted nanotape structure at pH 5 but (reversibly) a disordered state at pH 2.4. The images at pH 2.4 show possible fluctuations in density which may be due to the “correlation hole domains” detected by SAXS.

The role of π-stacking interactions in driving the self-assembly of the nanotape structure at pH 7 was probed using fluorescence experiments. The fluorescence spectra at pH 2.4 and pH 7 at an excitation wavelength λ_ex_ = 265 nm are shown in SI Figure S10. The red-shifted emission peak at 344 nm observed for pH 7 is absent at pH 2.4 (which shows a maximum at 294 nm), and is a signature of π-stacking of the pyridyl rings.^68^

Based on the cryo-TEM and SAXS data showing nanotape formation at pH 7, atomistic MD simulations of interdigitated β-sheets stacked in nanotapes were performed. The initial state was built as an array of antiparallel β-strands consistent with likely opposed arginine end groups, minimizing electrostatic interactions. SI Figure S11 shows the starting structure comprising four 32-mer β-sheets arranged into bilayer structures. The bilayers are built from fully interdigitated antiparallel molecules, consistent with the SAXS data analysis. Such an arrangement will also favor electrostatic interactions between the C-terminus of a peptide and the Arg residue of an adjacent molecule. The nanotape was observed to twist during the simulation run, consistent with the observations from cryo-TEM (Figure 3a), as shown in Figure 5. The equilibration of the system was monitored by plotting the solvent-accessible surface area (SASA) and related quantities (SI Figure S12). The simulations show that the hydrogen bond network was retained during the simulation run, as shown in Figure 5c and as confirmed by the plot of the number of hydrogen bonds during the run shown in SI Figure S13. The average number of hydrogen bonds per molecule remained around 4.3–4.6 during the run, showing that most amide groups are hydrogen-bonded. Ramachandran plot analysis (SI Figure S14) also confirmed that the β-sheet structure was stable during the simulation. It should be noted that changing the charge on the Pal_6_R, considering a charged N-terminus (net charge +1), led to unstable β-sheet nanosheets, which broke up into irregular clusters as shown by a representative configuration from a simulation run shown in SI Figure S14.

**Figure 5:**
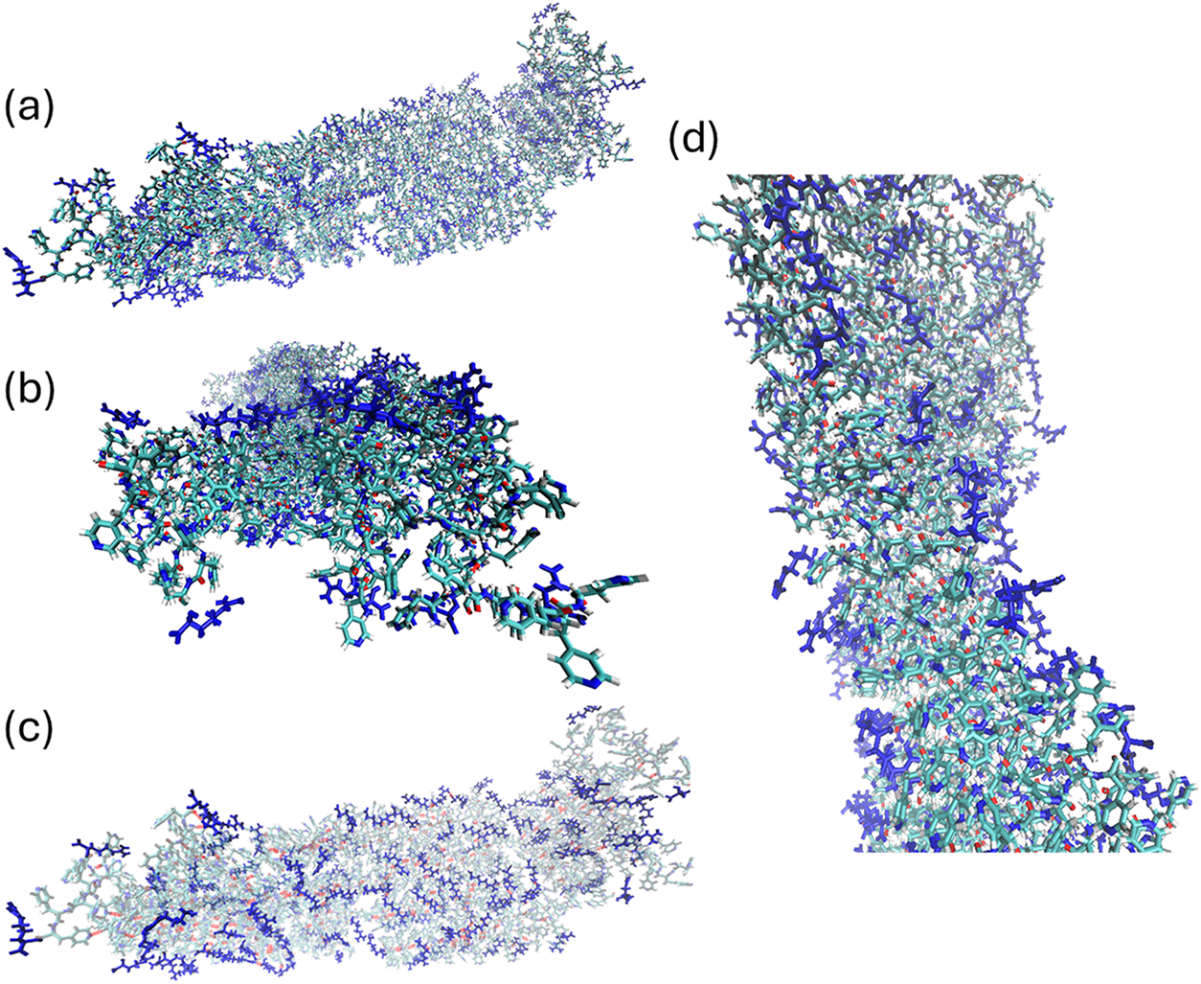
(a, b) Images of twisted nanosheet in different projections, observed at the end of an MD simulation run for Pal_6_R. (a) Side view, (b) end view. Arginine residues are colored deep blue. (c) Image showing an extensive array of hydrogen bonds (red dashed lines). (d) Enlarged view showing twisting within nanotape and arginine residues at the surface.

Due to its pH-dependent aggregation properties, we explored the use of Pal_6_R as a pH-responsive emulsion stabilizer. We selected the water-in-oil system using 1-bromohexadecane as the oil, closely density-matched to water to reduce potential sedimentation or creaming in unstable emulsions. We found that 0.1 wt % Pal_6_R at pH 7 is able to stabilize an emulsion for at least 24 h, as shown in Figure 6a, in contrast to the control mixture without the SLP. Optical microscopy images shown in Figure 6b,c show oil droplets with a polydisperse range of diameters from several ∼ μm to tens of μm. Laser scanning confocal microscopy (LSCM) was used to image the emulsion using ThT as a probe to locate peptide β-sheet structures. The images shown in Figure 6d–f reveal green fluorescent coatings of the oil droplets, indicating that the Pal_6_R β-sheets (stable at pH 7 in water, as discussed above) decorate the oil–water interface, stabilizing the emulsion. In contrast, at low pH 3, emulsion stabilization was not observed in the presence of Pal_6_R, as shown in SI Figure S16. The LSCM images show that the ThT fluorescence associated with β-sheets is not localized around the droplets, in contrast to the observations at pH 7 (Figure 6). This indicates that under conditions where the peptide molecules are highly charged, they do not assemble at the water/oil interface.

**Figure 6:**
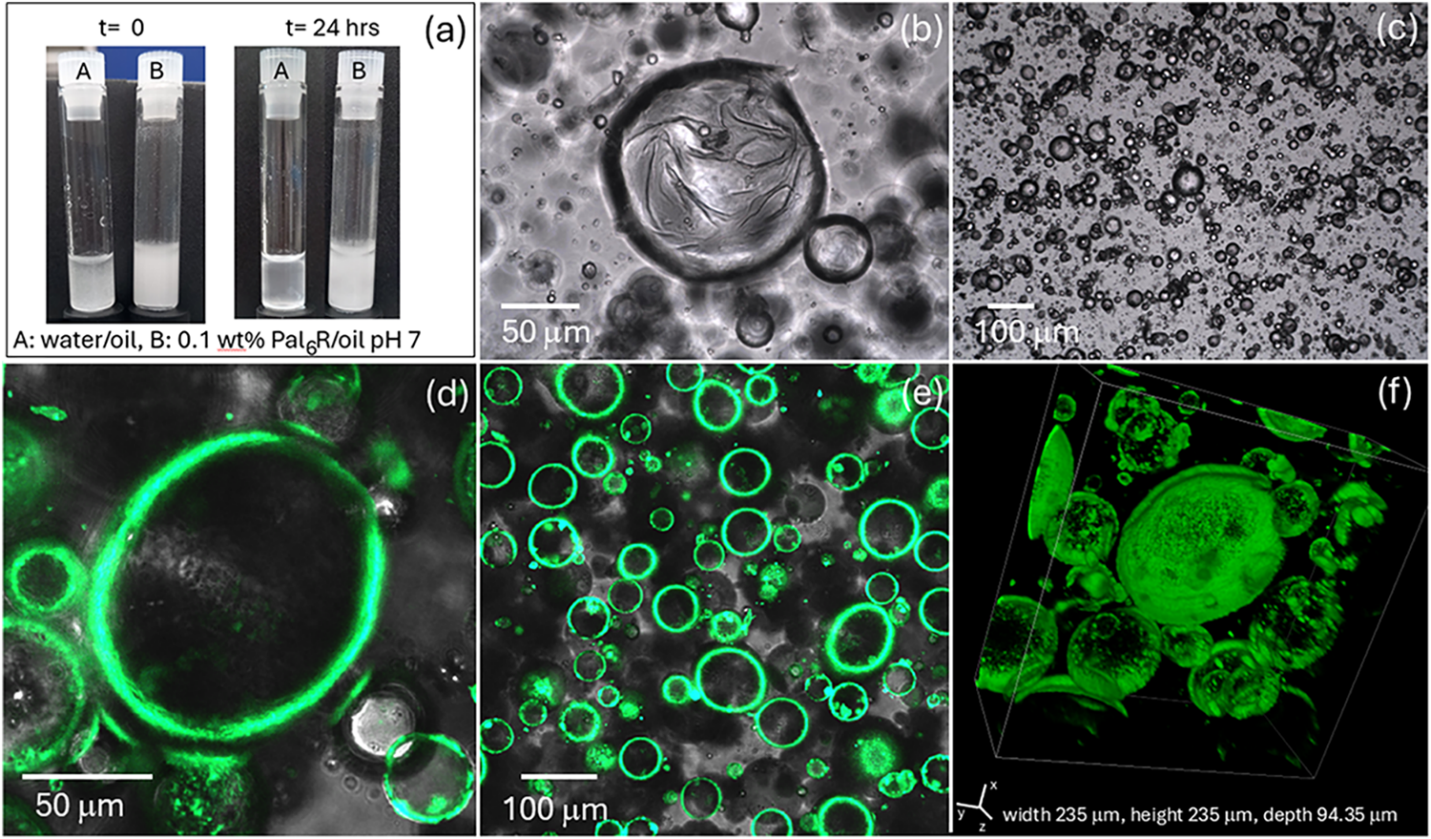
Water-in-oil emulsion stabilized by 0.1 wt % Pal_6_R at pH 7: (a) Stability over 24 h as compared to the 10:90 water:oil peptide-free emulsion. (b–c) Optical microscopy images. (d–f) LCSM images showing the ThT fluorescence for an emulsion stained with 5 × 10^–3^ wt % ThT (λ_ex_ = 488 nm). The images in parts (d, e) represent the overlap of the fluorescence of ThT with the transmission image.

The conformation of Pal_6_R in the emulsions with pH 4 and 7 was probed via CD spectroscopy. The CD data for the emulsion at low pH 4 (SI Figure S17a) is very similar to the CD spectrum for the Pal_6_R solution at pH 2.4 (Figure 2b). It is characteristic of a disordered structure with a contribution from the aromatic pyridine group (maximum at 219 nm). The spectrum for the Pal_6_R emulsion at pH 7 (SI Figure 17b) is very similar to the CD spectrum for the Pal_6_R solution at pH 7 (Figure 4b). It shows a strong negative minimum at 228 nm, assigned as a red-shifted β-sheet feature. The CD confirms that the β-sheet structure is retained in the emulsion. Self-assembly of Pal_6_R in the emulsions with pH 4 and 7 was probed via SAXS, the data being shown in SI Figure S17c. The SAXS data at pH 7 was analyzed using the same model used to fit the data for the peptide itself at this pH, whereas at pH 4, no peak was observed and only the sloping background was present. The fit parameters are listed in SI Table S3. At pH 7, the lamellar *d*-spacing is higher in the emulsion (*d* = 66.9 Å for an emulsion containing 0.1 wt % Pal_6_R, SI Table S3) than in the aqueous solution (*d* = 32.6 Å for 2 wt % Pal_6_R, SI Table S2). In addition, the peak is weaker and broader for the emulsion. This data supports the presence of bilayer nanotapes in the emulsion at pH 7, but not at pH 4. The bilayers are swollen in the emulsion, possibly due to the reduced concentration (i.e., higher water content) in the emulsion formulation, which also leads to fewer lamellar repeats than for 2 wt % Pal_6_R at pH 7 (Figure 3b, SI Table S2). Other possible explanations for the larger *d*-spacing in the emulsion include interface-induced reordering or changes in molecular packing in the lamellae.

## Conclusions

We investigated the pH-dependent conformation and association properties of the novel SLP Pal_6_R bearing the non-natural Pal (3-(4-pyridyl)-l-alanine) residue. Despite only comprising seven residues, at native pH 2.4, this peptide shows remarkable polyelectrolyte-like behavior. In particular, SAXS shows polyelectrolyte correlation hole scattering, with a correlation domain size scaling with concentration *d* ∼ *c* ^–(−0.277 ± 0.01)^ that is close to the theoretical predictions for semidilute salt-free solutions of weakly charged polyelectrolytes, *d* ∼ *c* ^–1/4^. The polyelectrolyte origin of the SAXS peak is confirmed by its absence in solutions containing NaCl, where the salt screens the repulsive charges on the peptide chains. To the best of our knowledge, this scaling behavior has not been reported previously on the basis of experimental data for any polyelectrolyte system. SAXS, SANS, and static light scattering data for several systems, including especially poly­(styrenesulfonate) show consistent *d* ∼ *c* ^–1/2^ scaling in semidilute solution with a crossover to *d* ∼ *c* ^–1/3^ in a more dilute solution.^51,69–71^ The data here indicate that Pal_6_R serves as a model system exhibiting close to the expected scaling for more weakly charged and flexible polyelectrolytes in semidilute salt-free solutions. The oligomeric nature of the peptide does not favor the rod-like conformation of conventional polyelectrolytes. It is believed that the weakly charged polyelectrolyte behavior results from the incomplete ionization of pyridyl residues. The p*K*_a_ values will significantly vary from the apparent values in practice due to the electrostatic effects from the arginine residue and terminal groups, and this may lead to only partial charging of particular pyridyl residues at pH 2.4. A full analysis would involve use of Poisson–Boltzmann theory to account for the electrostatics.

In addition to its notable polyelectrolyte properties, the pH responsiveness of the Pal_6_R sequence enables self-assembly at pH 7 into β-sheet-based bilayer nanotapes, as confirmed here using a combination of cryo-TEM, SAXS, XRD, and spectroscopic methods. The formation of twisted β-sheet nanotapes is also reproduced in MD simulations, which elucidate the conformational landscape and quantify the stabilization of the structures through extensive hydrogen bonding. We have previously shown that the analogue peptide A_6_R with uncapped termini forms arginine-coated nanosheets based on highly interdigitated bilayers in aqueous solution (which, at a high concentration, wrap into helical ribbons and nanotubes).^16^ The bilayer nanosheet formation is very similar to the behavior reported here, and this indicates that the nanostructure does not result specifically from the aromaticity of the Pal residues; it is due to the molecular amphiphilicity and the disposition of charged groups at the bilayer surfaces. This is further confirmed by the fact that the capped A_6_R peptide, i.e., CH_3_CO-A_6_R-NH_2_, forms cylindrical nanofibers instead of nanotapes/nanosheets.^12^

The designed SLP can stabilize water-in-oil emulsions at pH 7, i.e., under conditions where the peptide is expected to have a zero net charge, although with a zwitterionic-type structure comprising the Arg and adjacent C-terminus. The absence of net charge suggests that the emulsion is not charge-stabilized, and the presence of the β-sheet structure detected by confocal microscopy and CD spectroscopy suggests that the emulsion stabilization may arise from the surfactant-like nature of the peptide conferring interfacial activity and/or a Pickering-type steric stabilization mechanism. The layer packing of the peptide β-sheets is retained in the emulsion.

In summary, Pal_6_R is a valuable model system, which at low pH in aqueous solution is highly charged, leading to polyelectrolyte behavior corresponding to weakly charged flexible chains under semidilute conditions, with a correlation hole structure factor peak. The scaling of domain size with concentration for this class of polyelectrolyte has not previously been tested experimentally, although theory predicts a scaling relationship with which our data is in reasonable agreement. This surfactant-like peptide also shows remarkable pH-dependent conformational and self-assembly properties and, at pH 7, forms twisted β-sheet bilayer nanotapes. These structures can be used to stabilize water-in-oil emulsions at pH 7, which can be destabilized by reducing the pH to conditions where the β-sheet structures are absent.

## Supplementary Material



## Notes

Titration curve, additional cryo-TEM images, ANS fluorescence assay and original fluorescence spectra, polarized optical microscopy images, snapshots of structures from MD simulations, hydrogen bond numbers and Ramachandran plots from MD simulations, Images of sample vials and confocal microscopy for pH 4 Pal_6_R emulsions, CD and SAXS data for emulsions, and Tables of SAXS fit parameters (PDF)
