# Supplementary material for: Polyelectrolyte-like Behavior, pH-Dependent Self-Assembly, and Emulsion Stabilizing Properties of a Model Surfactant-like Peptide Bearing Pyridine Groups
Source: Biomacromolecules. 2026 Jun 11;27(7):4571–80. doi: 10.1021/acs.biomac.6c00476 (PMC13370784; doi:10.1021/acs.biomac.6c00476)
Supplement: Supplementary file 1 [file bm6c00476_si_001.pdf]

Supporting Information

Polyelectrolyte-Like Behavior, pH-Dependent Self-Assembly and Emulsion Stabilizing Properties of a Model Surfactant-Like Peptide Bearing Pyridine Groups

Valeria Castelletto, [1] Jani Seitsonen,[2] Ian W Hamley[1,*]

[1] *School of Chemistry, Food Biosciences and Pharmacy, University of Reading, Whiteknights, Reading RG6 6AD, U.K.*

[2] *Nanomicroscopy Center, Aalto University, Puumiehenkuja 2, FIN-02150 Espoo, Finland*

* Author for correspondence, I.W.Hamley@reading.ac.uk

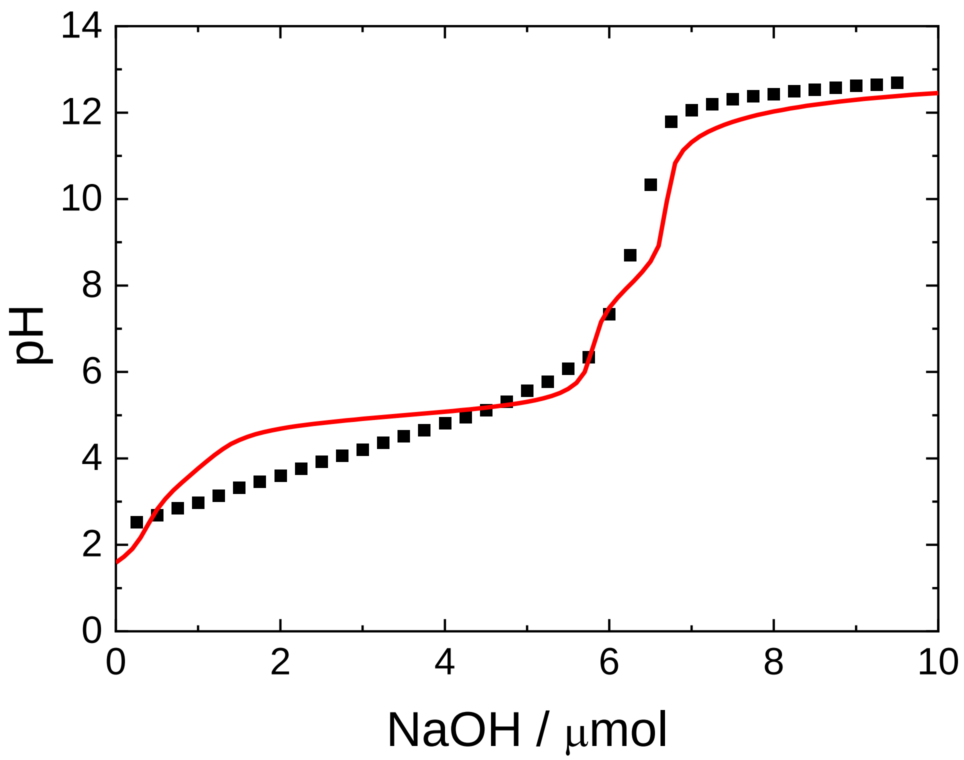

SI Fig S1. Measured titration curve for 1.2 wt% Pal$_6$R (open symbols) along with calculated titration curve using Henderson-Hasselbalch equation (red line) assuming pKa(Arg, side chain) = 12, pKa(N terminus) = 8, pKa(3-pyridyl) = 5, pKa(C terminus) = 3.5.

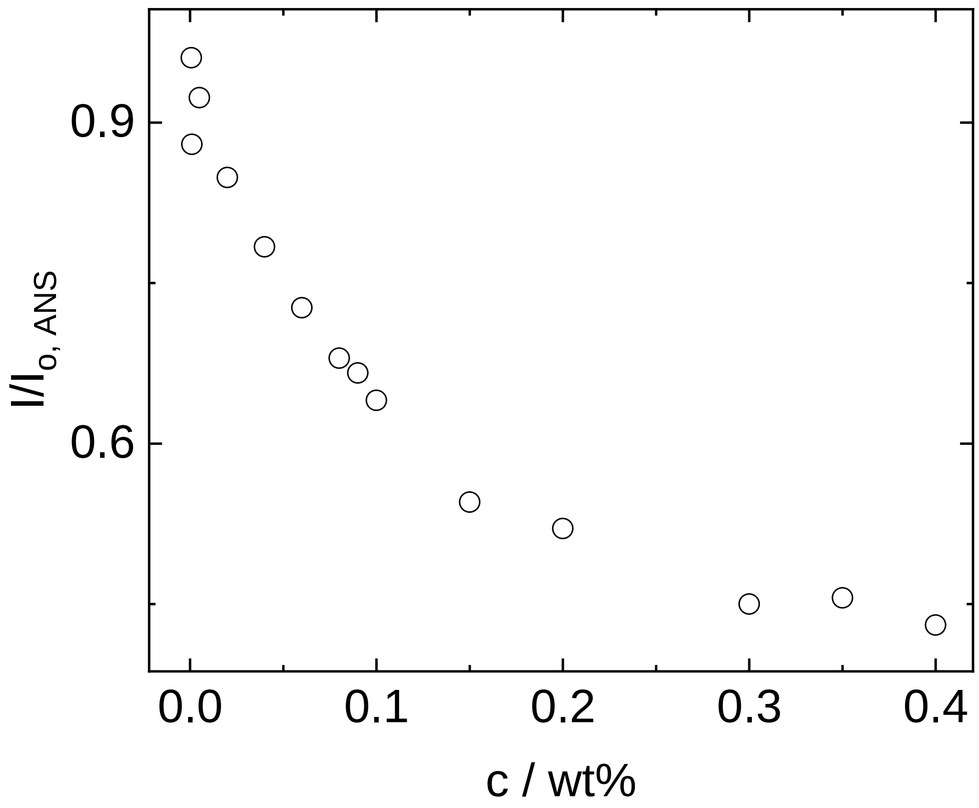

SI Fig.S2. ANS peak fluorescence I relative to that of solution without peptide $I_{0,ANS}$ for native pH 2.4 solutions of Pal_6R.

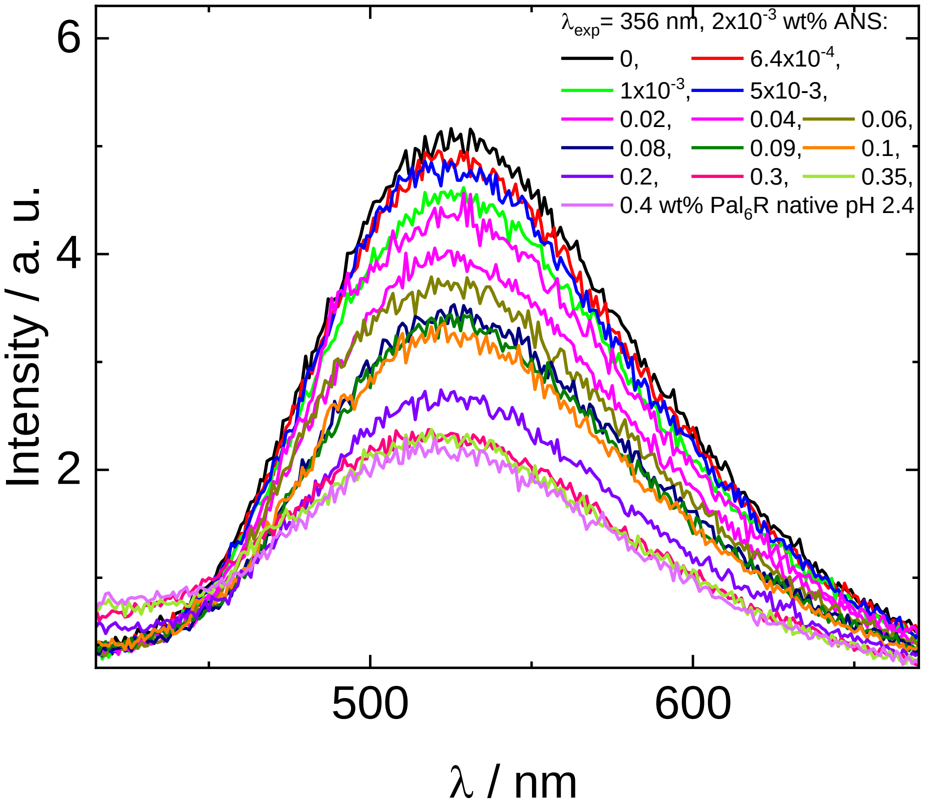

SI Fig.S3. Fluorescence spectra for ANS + Pal_6R native pH 2.4 at the concentrations (wt%) indicated.

(a)

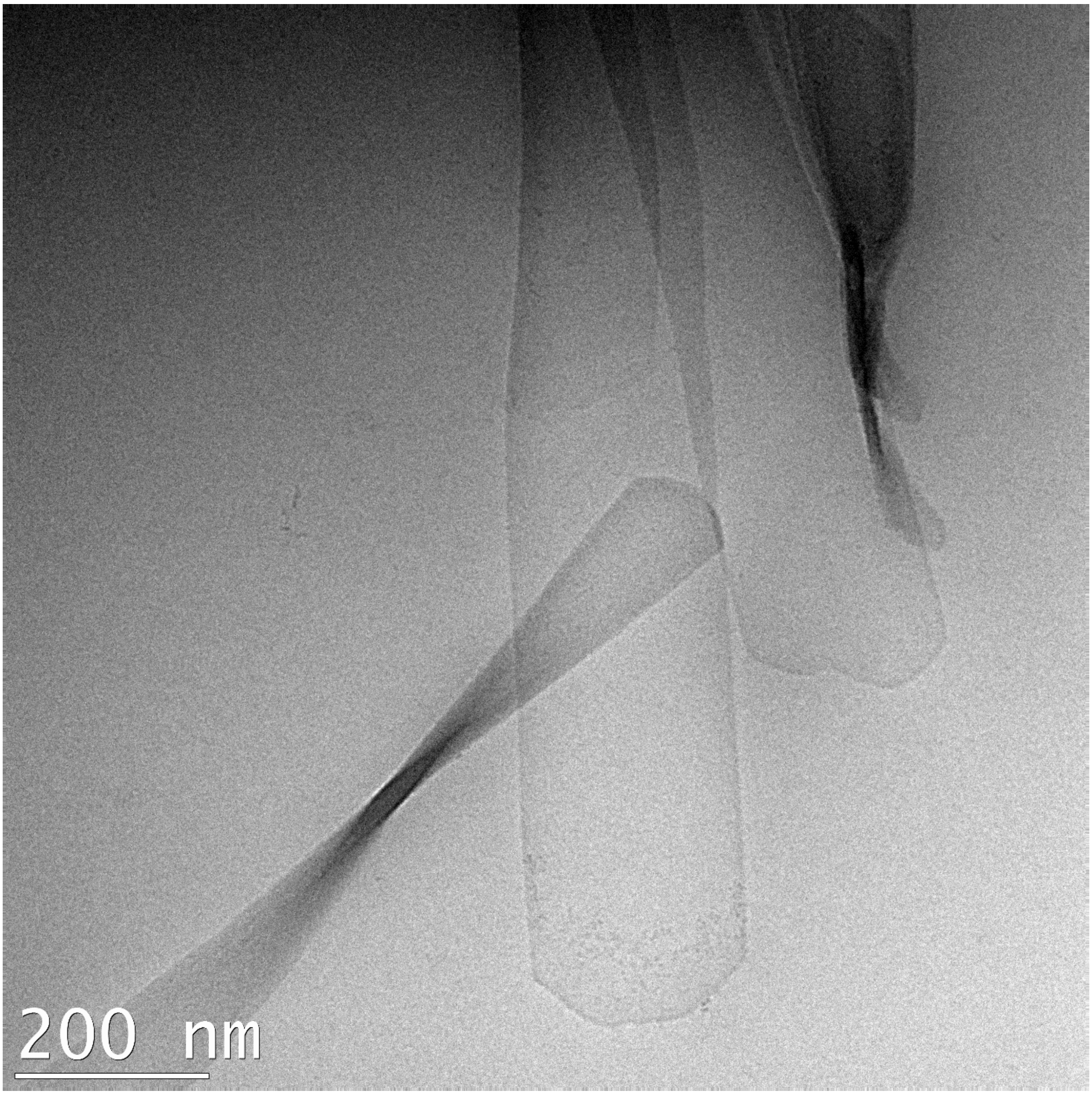

(b)

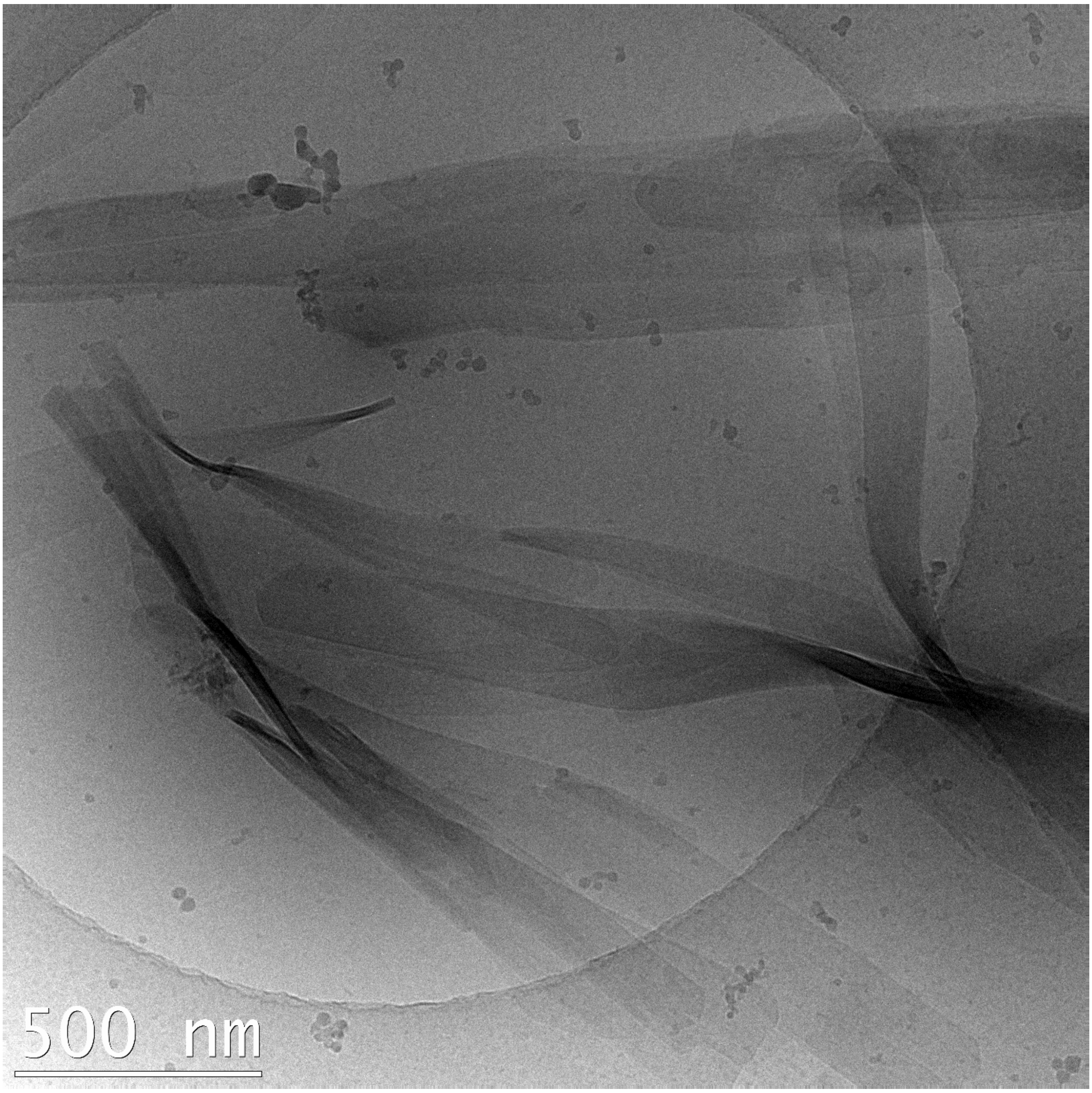

SI Fig.S4. (a,b) Additional cryo-TEM images for 1 wt% solution of Pal_6R at pH 7.

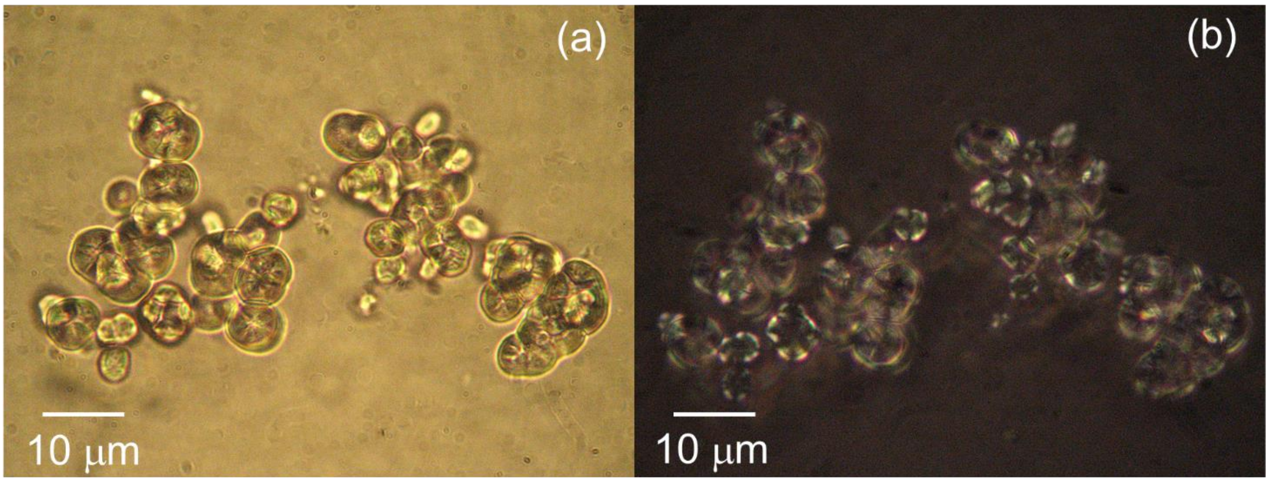

SI Fig S5. Polarized optical microscopy images for a 2 wt% solution of Pal_6R at pH 7, (a) bright field, (b) Crossed polarizers.

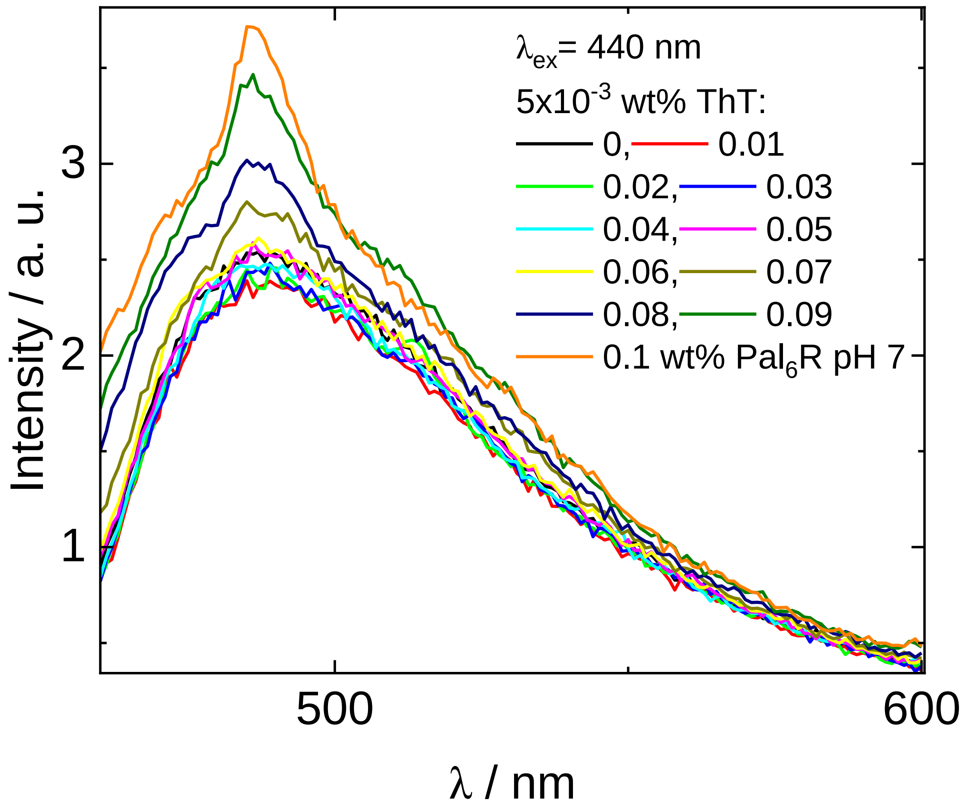

SI Fig.S6. Fluorescence spectra for ThT + Pal$_6$R pH 7 at the concentrations (wt%) indicated.

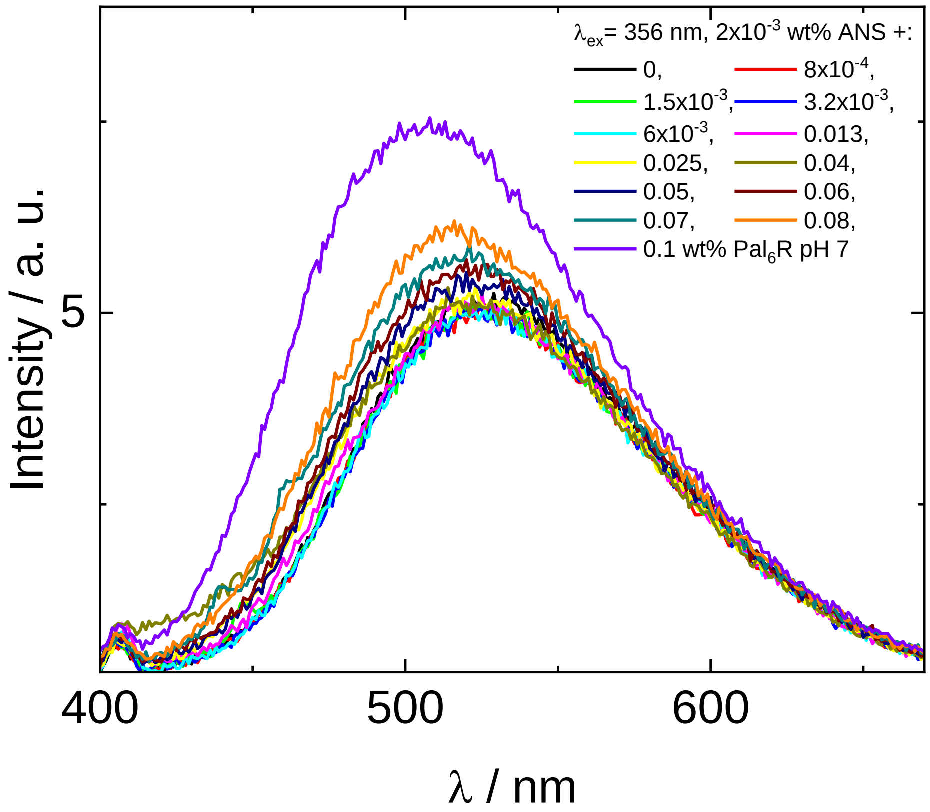

SI Fig.S7. Fluorescence spectra for ANS + Pal_6R pH 7 at the concentrations (wt%) indicated.

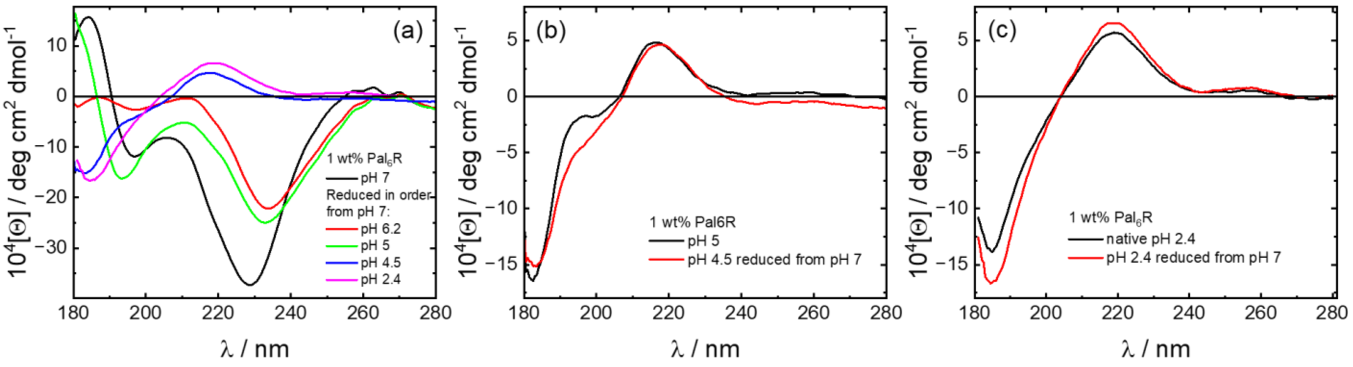

SI Fig.S8. pH-dependent CD spectra for 1 wt% Pal_6R. (a) Spectra obtained at the pH values indicated, starting from pH 7. (b) Comparison of spectra at pH 4.5 and pH 5 for samples measured directly at this pH value or for a sample prepared by pH reduction from pH 7, (c) Comparison of spectra at pH 2.4 for samples measured directly at this pH value or for a sample prepared by pH reduction from pH 7. Samples above pH 5 were observed to be cloudy due to the formation of extended β-sheet nanotapes.

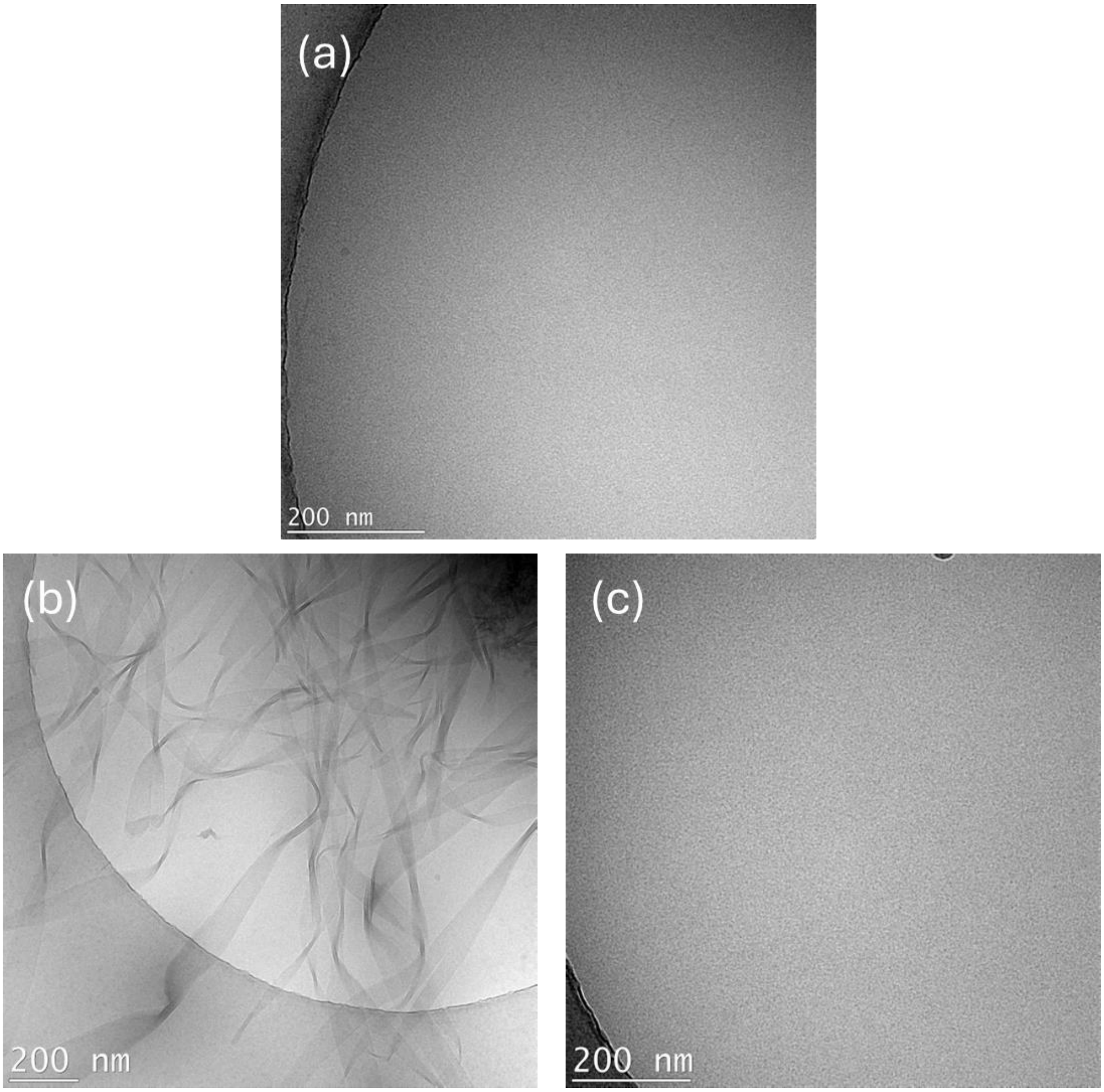

SI Fig.S9. Cryo-TEM images for samples at different pH (including reversibility study). (a) 2 wt% Pal_6R at pH 2.4, (b) 1 wt% Pal_6R at pH 5, (c) 1 wt% Pal_6R with pH reduced from pH 5 to native pH 2.4.

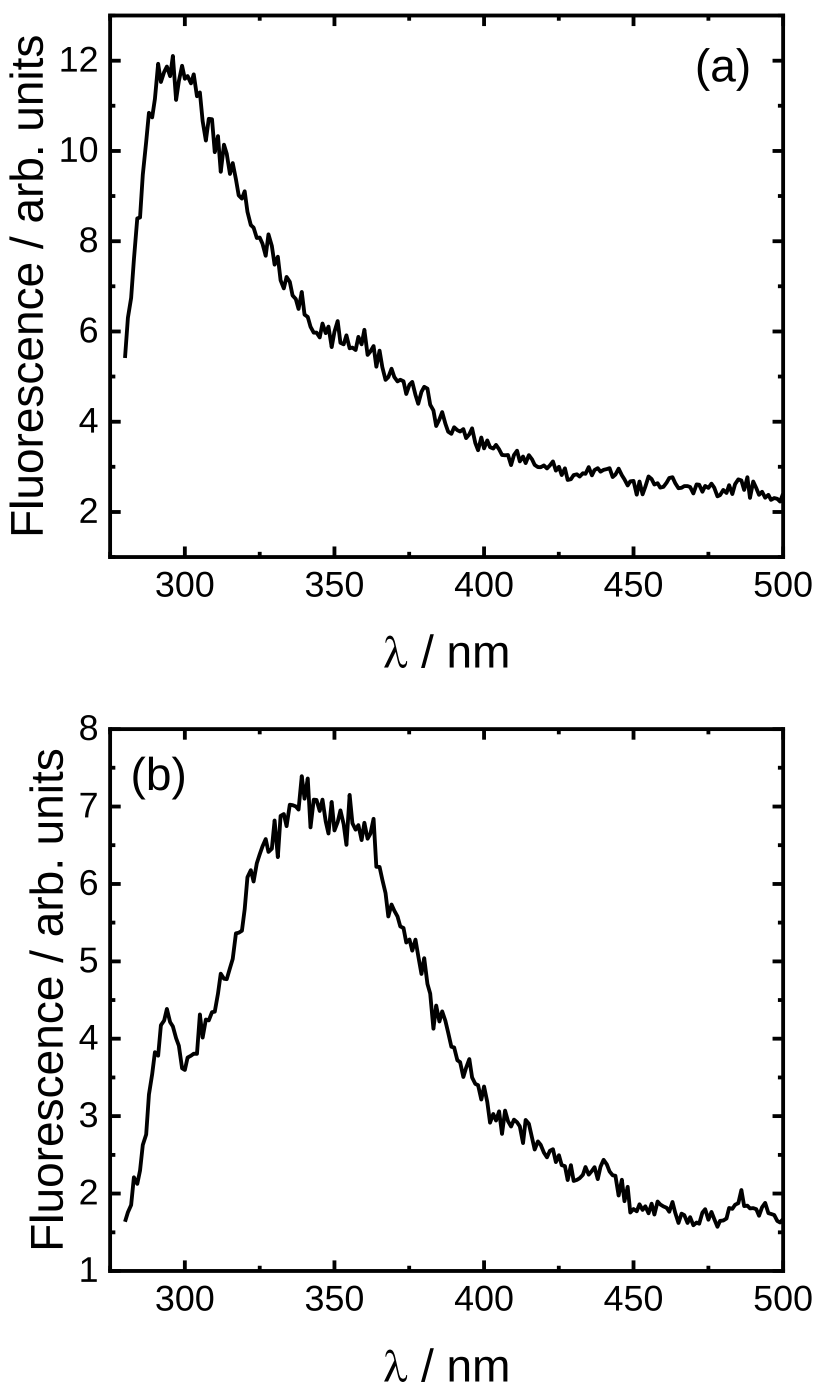

Fig.S10. Fluorescence spectra for Pal_6R 0.1 wt% solutions with λ_{ex} = 265 nm. (a) pH 2.4, (b) pH 7.

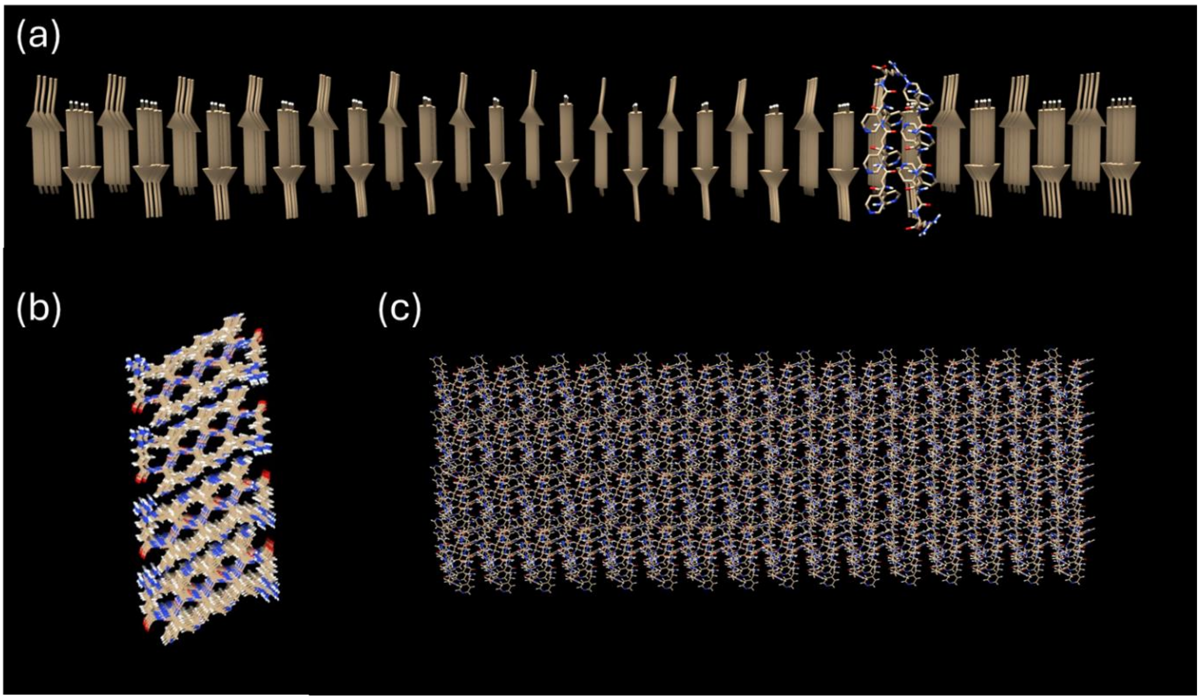

SI Fig.S11. Initial configuration of 128 molecules of Pal$_6$R in antiparallel β-sheet nanotape of structure for MD simulations. (a) Side view in cartoon format with explicit representation of molecules for one dimer pair, (b) End view, (C) Top view.

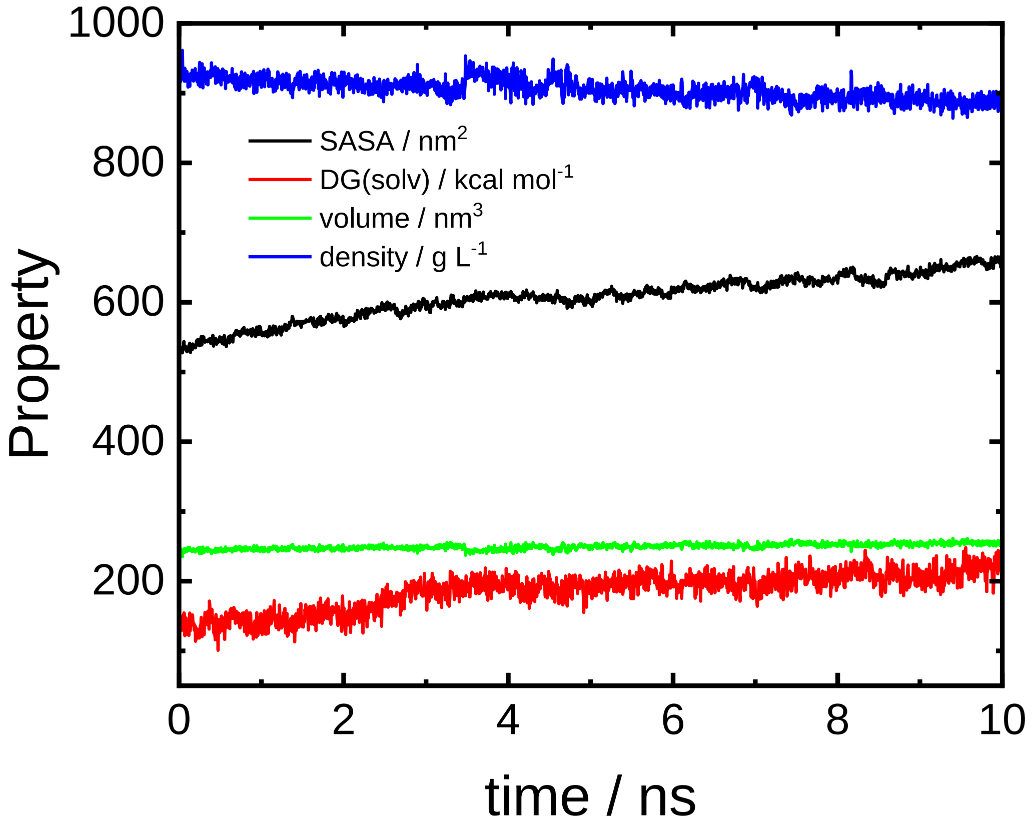

SI Fig.S12. Solvent accessible surface area (SASA) and related quantities shown, during simulation run for 128 molecule antiparallel β-sheet nanotape of Pal_6R.

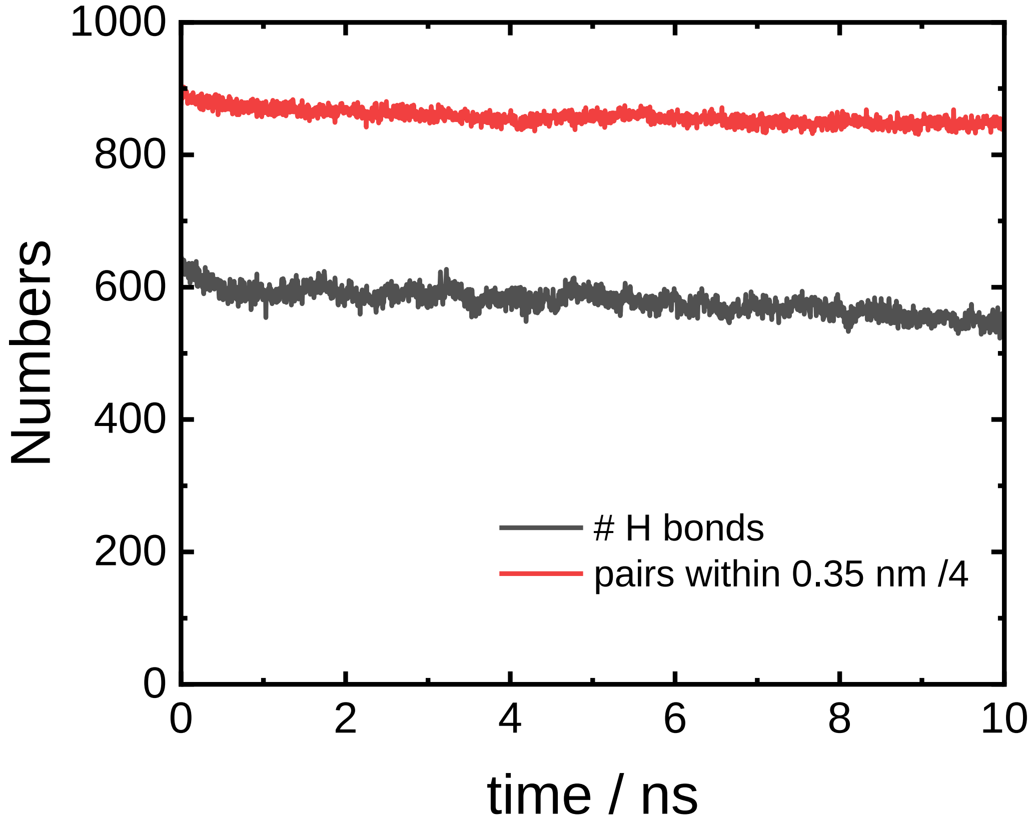

SI Fig.S13. Number of hydrogen bonds and number of H-bond capable pairs within 0.35 nm (scaled by ¼ for ease of visualization) during simulation run for 128 molecule antiparallel β-sheet nanotape of Pal_6R.

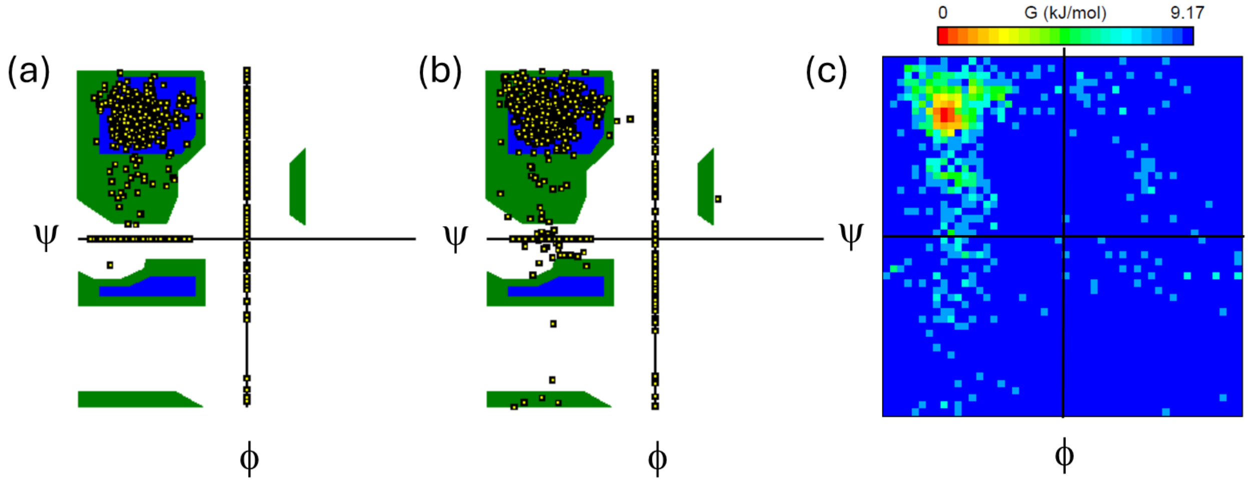

SI Fig.S14. Ramachandran plots from MD simulations of 128 molecule β-sheet nanotape of Pal$_6$R. (a) Initial, (b) Final (after 10 ns). The backbone angles for all residues in all molecules are plotted as yellow points. (c) Final frame Ramachandran plot in terms of Gibbs free energy.

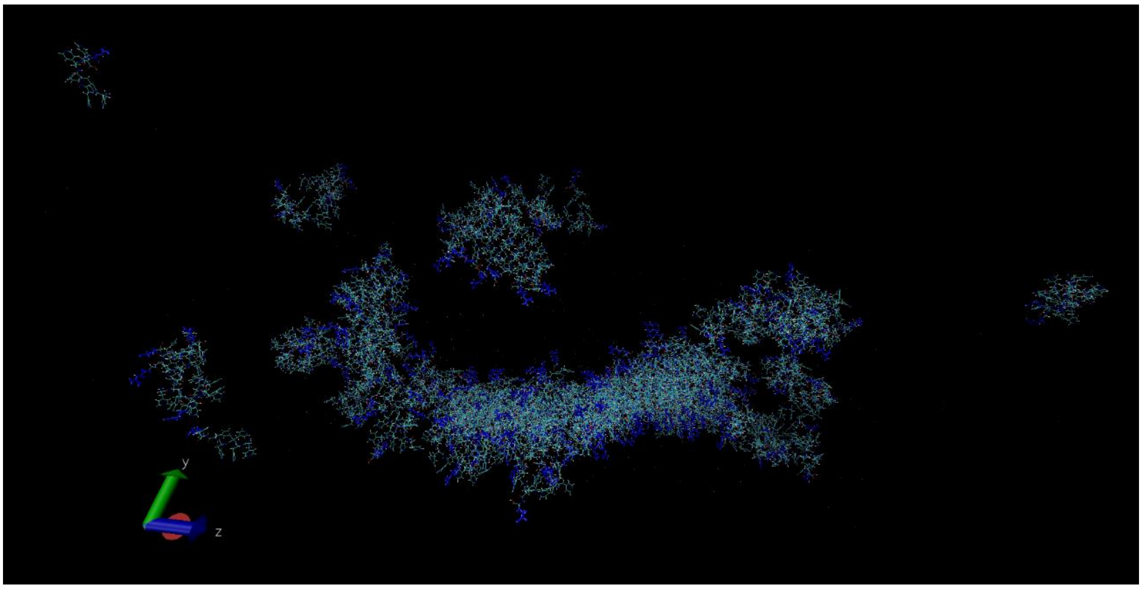

SI Fig.S15. View from final frame of MD simulation for Pal$_6$R with charge +1 started from initial 256-mer β-sheet nanotape structure showing breakup into micelle-like clusters. Arginine residues are coloured deep blue.

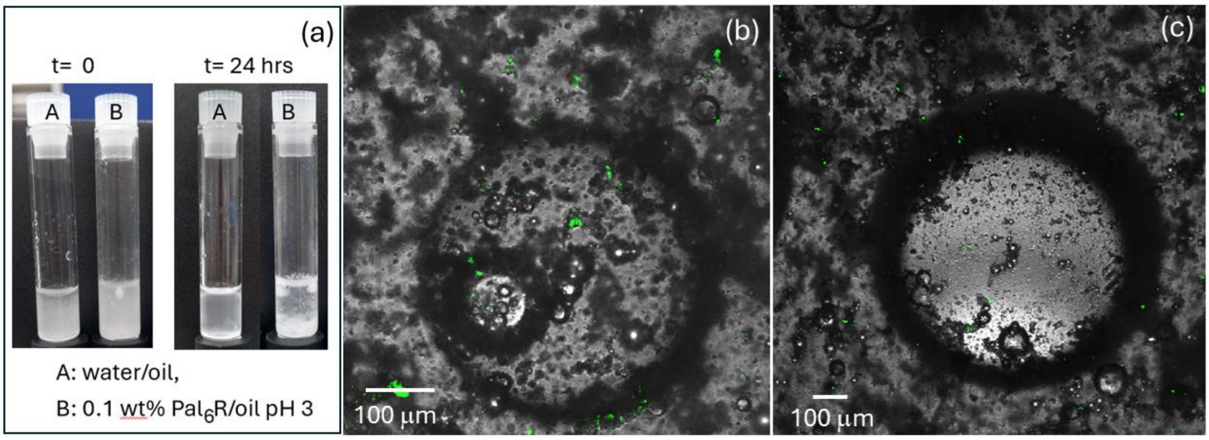

SI Fig. S16. Emulsion instability for 0.1 wt% Pal$_6$R at pH 4. (a) Stability over 24 hours as compared to the 10:90 water:oil peptide-free emulsion. (b, c) LSCM images for the emulsion stained with 5×10^{-3} wt% ThT (λ_{ex} = 488 nm). The images in parts (b, c) represent the overlap of the fluorescence of ThT with the transmission image.

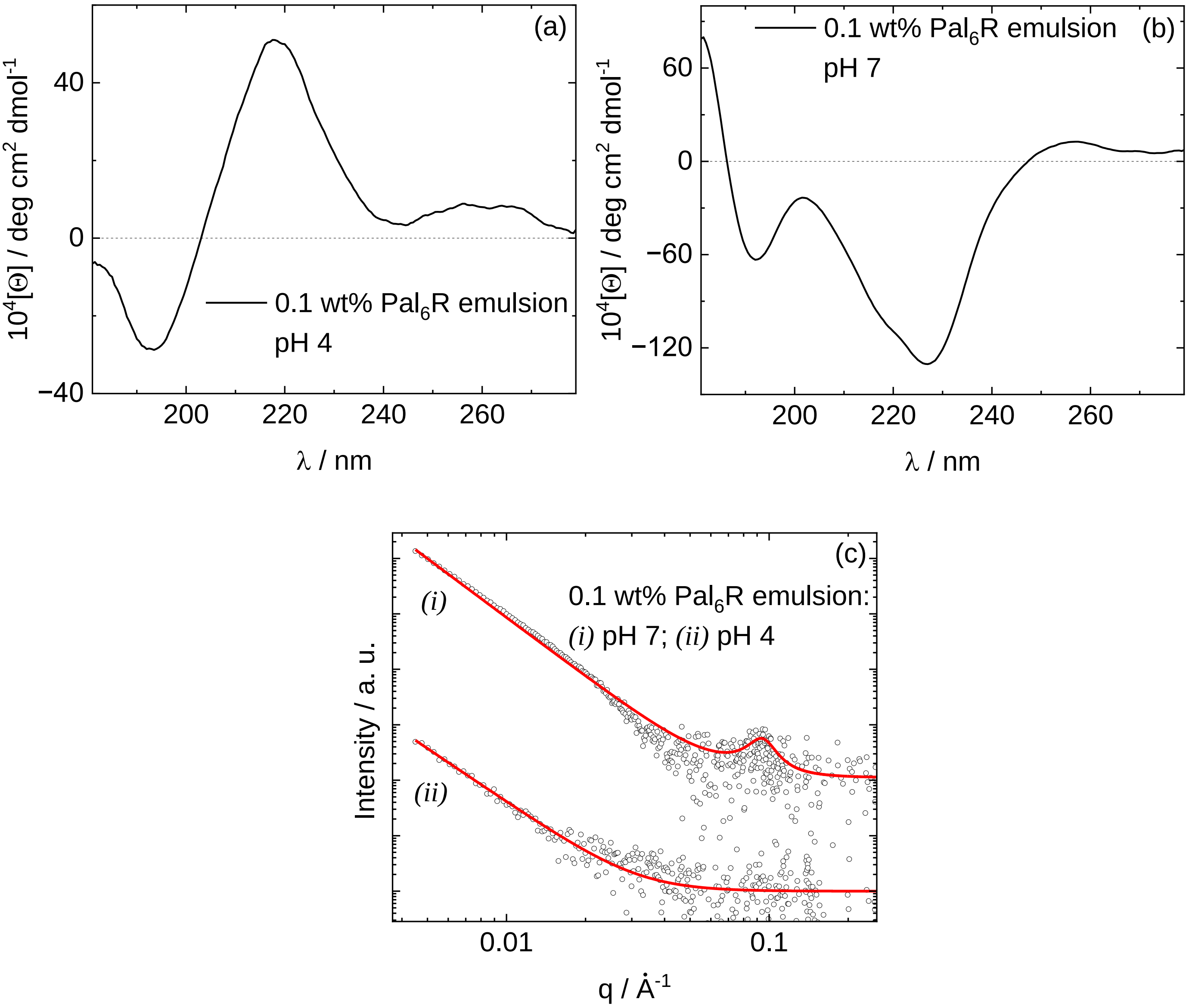

SI Fig. S17. CD and SAXS data 0.1 wt % Pal$_6$R emulsions. CD spectra for emulsions at (a) pH 4 and (b) pH 7, (c) SAXS for emulsions at pH 4 or pH 7, open symbols: measured data, solid red lines: fitted intensity profiles as described in the text (fit parameters in SI Table S3). For ease of visualization only every second data point is shown and in (*ii*) data has been shifted vertically.

Table S1. Parameters extracted from the fitting of the SAXS data in Fig. 1b for Pal_6R at native pH 2.4. Fitted using SASfit.[1-2]

	1 wt% Pal_6R	2 wt% Pal_6R	3 wt% Pal_6R	4 wt% Pal_6R	5 wt% Pal_6R	6 wt% Pal_6R
A / cm^{-1}	6.61×10^{-3}	1.43×10^{-2}	1.95×10^{-2}	2.37×10^{-2}	2.87×10^{-2}	3.30×10^{-2}
q^* / $Å^{-1}$	0.0838	0.1032	0.1165	0.1243	0.1318	0.1375
w / $Å^{-1}$	0.104	0.102	0.100	0.101	0.104	0.107
B / cm^{-1}	1.90×10^{-3}	2.63×10^{-2}	4.63×10^{-2}	6.61×10^{-2}	7.11×10^{-2}	6.17×10^{-2}
I_0 / cm^{-1} §	1.00×10^{-4}	1.00×10^{-4}	1.00×10^{-4}	1.00×10^{-4}	1.00×10^{-4}	1.00×10^{-4}
I_s / cm^{-1}	-7.32×10^{-4}	-2.42×10^{-2}	-4.42×10^{-2}	-6.44×10^{-2}	-7.02×10^{-2}	-6.19×10^{-2}
α	0.247	0.0083	-0.0157	-0.0240	-0.0378	-0.0631

Key: Lorentzian Peak: peak amplitude; **A**, peak centre, $\mathbf{q^*}$; width of Lorentzian function, **w**; background **B**. **Sloping background**: $I = I_0 + I_s q^{-\alpha}$. § fixed parameter.

Table S2. Parameters extracted from the fitting of the SAXS data in Fig. 3b for Pal_6R at pH 7 and pH 12. Fitted using SASfit.[1-2]

	2 wt% Pal_6R pH 7	2 wt% Pal_6R pH 12
A / cm^{-1}	1.1	3.8
q_0 / $Å^{-1}$	0.195	0.201
w / $Å^{-1}$	1.1×10^{-2}	4.4×10^{-3}
B / cm^{-1}	1.02	0.98
I_o / cm^{-1}	1.6×10^{-3}	1.9×10^{-3}
I_s / cm^{-1}	2.92×10^{-7}	3.2×10^{-7}
α	3.73	3.68

Key: Lorentzian Amplitude Structure Factor: peak amplitude; **A**, peak centre, $\mathbf{q_0}$; width of Lorentzian function, **w**; background **B**. **Sloping background**: $I = I_0 + I_s q^{-\alpha}$.

Table S3. Parameters extracted from the fitting of the SAXS data in SI Fig.S17 for emulsions containing 0.1 wt% Pal_6R at pH 4 and pH 7. Fitted using SASfit.[1-2]

	0.1 wt% Pal_6R emulsion pH 4	0.1 wt% Pal_6R emulsion pH 7
A / cm^{-1}	--	2.3
q_0 / $Å^{-1}$	--	0.0939
w / $Å^{-1}$	--	0.012
B / cm^{-1}	--	0.74
I_o / cm^{-1}	$9.9x10^{-4}$	$9.9x10^{-4}$
I_s / cm^{-1}	$1.6x10^{-8}$	$6.0x10^{-7}$
α	3.21	3.54

Key: Lorentzian Amplitude Structure Factor: peak amplitude; **A**, peak centre, $\mathbf{q_0}$; width of Lorentzian function, **w**; background **B**. **Sloping background**: $I = I_0 + I_s q^{-\alpha}$.

References

(1) Bressler, I.; Kohlbrecher, J.; Thünemann, A. F., SASfit: a tool for small-angle scattering data analysis using a library of analytical expressions. *J. Appl. Cryst*. **2015,** *48*, 1587–1598.

(2) Kohlbrecher, J.; Bressler, I., Updates in SASfit for fitting analytical expressions and numerical models to small-angle scattering patterns. *J. Appl. Cryst*. **2022,** *55*, 1677–1688.
